# Assessing Professionalism in Medicine – A Scoping Review of Assessment Tools from 1990 to 2018

**DOI:** 10.1177/2382120520955159

**Published:** 2020-10-16

**Authors:** Kuang Teck Tay, Shea Ng, Jia Min Hee, Elisha Wan Ying Chia, Divya Vythilingam, Yun Ting Ong, Min Chiam, Annelissa Mien Chew Chin, Warren Fong, Limin Wijaya, Ying Pin Toh, Stephen Mason, Lalit Kumar Radha Krishna

**Affiliations:** 1 Yong Loo Lin School of Medicine, National University of Singapore, Singapore; 2 National University Hospital, National University Health System, Singapore; 3 School of Medicine, International Medical University Malaysia, Kuala Lumpur, Malaysia; 4 Division of Cancer Education, National Cancer Centre Singapore, Singapore; 5 Medical Library, National University of Singapore Libraries, National University of Singapore, Singapore; 6 Department of Rheumatology and Immunology, Singapore General Hospital, Singapore; 7 Department of Infectious Diseases, Singapore General Hospital, Singapore; 8 Department of Family Medicine, National University Health System, Singapore; 9 Palliative Care Institute Liverpool, Academic Palliative & End of Life Care Centre, University of Liverpool, Liverpool, UK; 10 Division of Supportive and Palliative Care, National Cancer Centre Singapore, Singapore; 11 Centre for Biomedical Ethics, National University of Singapore, Singapore; 12 Duke-NUS Graduate Medical School, Singapore; 13 PalC, The Palliative Care Centre for Excellence in Research and Education

**Keywords:** Medicine, professionalism, medical school, physicians, medical professionalism, assessment methods, medical education

## Abstract

**Background:**

Medical professionalism enhances doctor-patient relationships and advances patient-centric care. However, despite its pivotal role, the concept of medical professionalism remains diversely understood, taught and thus poorly assessed with Singapore lacking a linguistically sensitive, context specific and culturally appropriate assessment tool. A scoping review of assessments of professionalism in medicine was thus carried out to better guide its understanding.

**Methods:**

Arksey and O’Malley’s (2005) approach to scoping reviews was used to identify appropriate publications featured in four databases published between 1 January 1990 and 31 December 2018. Seven members of the research team employed thematic analysis to evaluate the selected articles.

**Results:**

3799 abstracts were identified, 138 full-text articles reviewed and 74 studies included. The two themes identified were the context-specific nature of assessments and competency-based stages in medical professionalism.

**Conclusions:**

Prevailing assessments of professionalism in medicine must contend with differences in setting, context and levels of professional development as these explicate variances found in existing assessment criteria and approaches. However, acknowledging the significance of context-specific competency-based stages in medical professionalism will allow the forwarding of guiding principles to aid the design of a culturally-sensitive and practical approach to assessing professionalism.

## Background

Medical professionalism underpins the development of trusting doctor-patient relationships that help inform and guide the delivery of socioculturally sensitive, patient-centric care and enhance healthcare outcomes and overall patient satisfaction.^1-4^ It forms the cornerstone of effective self-regulation and the promulgation of a transparent, accountable and evidence-based clinical practice.^1-3^ Ensuring that medical professionalism is effectively practised is thus pivotal to the standing of the medical profession, the preservation of public trust and the provision of quality healthcare.^5-8^

However, the perception of healthcare services as commodities subjected “*to the forces of commercialisation and profit-making in the free-market economy*”^9^ has seen the erosion of professional, moral and ethical values in medical practice. In addition to this scrupulous emphasis on productivity, the profession has struggled to “*hold up against [the] prevailing philosophy of moral scepticism and relativism in the present post-modernist society*”.^9^ Pervasive technological changes to the therapeutic relationship has also led to the blurring of professional boundaries between doctors and patients^10-12^ and these issues have complicated oversight of appropriate professional behaviour in medicine.^11,13^ These considerations underscore the need to better assess medical professionalism.^14-17^

## Defining medical professionalism

A consistent understanding of medical professionalism remains elusive in part due to the influence of practical and local sociocultural, legal, financial, educational and healthcare considerations.^3,18-30^ This diversity is captured in Birden, et al’s^31^ comprehensive overview of key concepts in professionalism and in the definitions/domains of medical professionalism proffered by the Accreditation Council for Graduate Medical Education (ACGME)^32^ in the United States, the General Medical Council (GMC)^33^ in the United Kingdom and the Canadian Medical Education Directives for Specialists (CanMEDS)^34^ (Table 1).

**Table 1.** Definition/domains of medical professionalism.

Healthcare regulatory organisations	Definitions/domains of medical professionalism
Accreditation Council for Graduate Medical Education (ACGME)	(a) Demonstrating professional conduct and accountability. (b) Demonstrating humanism and cultural proficiency. (c) Maintaining emotional, physical, and mental health. (d) Pursuing continual personal and professional growth.
General Medical Council (GMC)	The domains for professionalism include: (a) Knowledge, skills, and performance • Make the care of your patient your first concern. • Provide a good standard of practice and care. (b) Safety and quality • Take prompt action if you think that patient safety, dignity or comfort is being compromised. • Protect and promote the health of patients and the public. (c) Communication partnership and teamwork • Treat patients as individuals and respect their dignity. • Work in partnership with patients. • Work with colleagues in the ways that best serve patients’ interests. (d) Maintaining trust • Be honest and open and act with integrity. • Never discriminate unfairly against patients or colleagues. Never abuse your patients’ trust in you or the public’s trust in the profession.
Canadian Medical Education Directives for Specialists (CanMEDS)	As Professionals, physicians are committed to the health and well-being of individual patients and society through ethical practice, high personal standards of behaviour, accountability to the profession and society, physician-led regulation, and maintenance of personal health. • Commitment to patients • Commitment to society • Commitment to the profession • Commitment to self

Recognising this diversity in concepts, the Ottawa Consensus Group meeting in 2018 acknowledged the need to explore “*the perspectives of patients and the tensions of individual and institutional values in regard to professionalism*” as opposed to “*a standardised, single, and reductionist definition*”.^2^ This position underlines the need for context-specific, culturally appropriate and linguistically sensitive assessments.^1,2^

## The need for this review

The absence of such a context-specific, culturally appropriate and linguistically sensitive assessment tool within the Singapore setting inspired this scoping review. It is hoped that the insights proffered will guide design of a tool for the local context.

## Methods

Building upon Li et al’s,^35^ Wilkinson et al’s^4^ and Veloski et al’s^36^ reviews, this scoping review explores the size and scope^37^ of published data in peer-reviewed literature on tools used to assess medical professionalism.^38-43^ Use of a scoping review also allows for the systematic extraction, synthesis and summarising of actionable and applicable information^44^ in available literature^45,46^ across a wide range of pedagogies, assessment contents and practice settings.^47-51^ Levac et al’s^52^ adaptation of Arksey and O’Malley’s^38^ framework for scoping reviews was used to map key concepts, sources and types of evidences available to guide future research.^39,53,54^ Levac et al’s framework was also guided concurrently by the PRISMA-P 2015 checklist^45^ and a 6-stage scoping review protocol was developed for this study.^38-42,52^

### Stage 1: Identifying the research question

The 11-member research team was guided by a team of local clinicians, educators and researchers from Yong Loo Lin School of Medicine at National University of Singapore (YLLSoM), Duke-NUS Medical School, the University of Liverpool and the National Cancer Centre Singapore (NCCS), and two librarians from the medical libraries at YLLSoM and NCCS (henceforth the expert team). The expert and research teams discussed prevailing issues, needs, and practices pertaining to assessment tools used in medical professionalism and determined the primary research question to be: ‘what tools are available to assess medical professionalism among medical students or physicians?’ The teams determined the secondary research questions to be: ‘what domains are assessed and what approaches are used to carry out these assessments?’ and ‘what existing frameworks are used to guide them?’ These questions were designed based on prevailing population, conceptual and contextual considerations^55^ using a PICOS format (Table 2).

**Table 2.** PICOS, inclusion and exclusion criteria.

PICOS	Inclusion criteria	Exclusion criteria
Population	• Undergraduate and postgraduate medical students, residents or doctors within the clinical, medical, research or academic settings	• Allied health specialties such as Pharmacy, Dietetics, Chiropractic, Midwifery, Podiatry, Speech Therapy, Occupational and Physiotherapy • Non-medical specialties such as Clinical and Translational Science, Alternative and Traditional Medicine, Veterinary, Dentistry
Intervention	• Curricular assessment tools on professionalism of medical students or doctors	
Comparison	• Comparisons of the various assessment tools, including the assessment principles, modalities and criteria	
Outcome	• Assessment principles, modalities, and criteria • Impact of assessment on those being assessed	
Study design	• Articles in English or translated to English • All study designs including: ○ Mixed methods research, meta-analyses, systematic reviews, randomised controlled trials, cohort studies, case-control studies, cross-sectional studies, and descriptive papers • Year of Publication: 1 January 1990 to 31 December 2018 • Databases: PubMed, ERIC, Google Scholar, Web of Science databases	• Grey Literature/electronic and print information not controlled by commercial publishing • Case reports and series, ideas, editorials, and perspectives

These collaborative consultations between the research and expert teams also took place at Stage 1, 2, 3 and 6 of the research process.

### Stage 2: Identifying relevant studies

Guided by the expert team and prevailing definitions and descriptions of medical professionalism by ACGME, GMC and CanMEDS,^32,33,56^ the research team developed their search strategy which may be found in Online Appendix A.

In keeping with Pham et al’s^54^ approach to ensuring a viable and sustainable research process, the research team confined the searches to articles published between 1 January 1990 and 31 December 2018 on PubMed, ERIC, Web of Science and Google Scholar. All qualitative and quantitative research methodologies in peer-reviewed articles published in English or had English translations were included. The independent searches of the four databases were carried out between 13 February 2019 and 24 April 2019.

### Stage 3: Selecting studies to be included in the review

The nine members of the research team independently screened the titles and abstracts by exporting and organising the articles using the Endnote software. This allowed for the removal of duplicate titles and the generation of a tentative list. Sandelowski and Barroso’s^57^ ‘negotiated consensual validation’ approach was used to agree on the final list of articles to be reviewed and, later, the final list of full text articles to be scrutinised.

**Figure 1.**
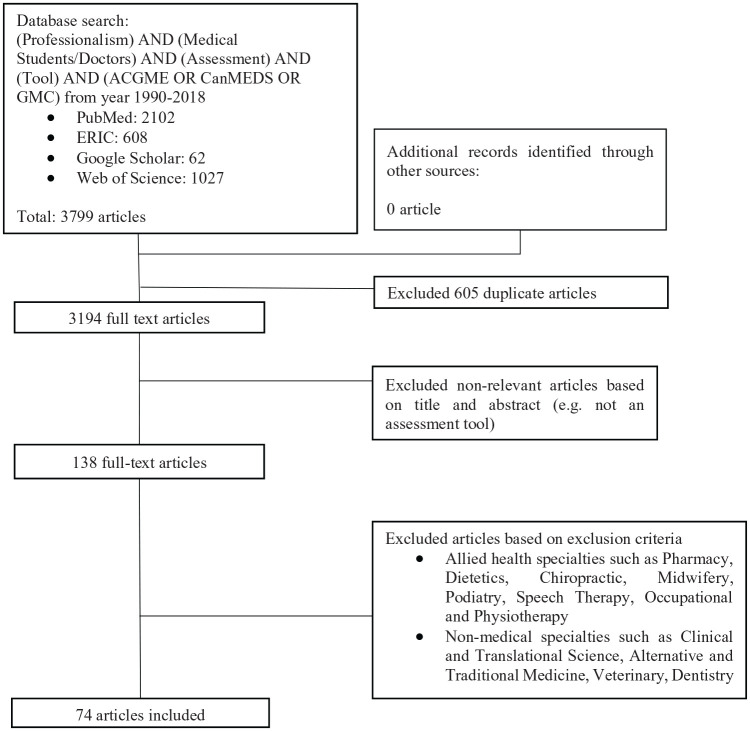
PRISMA flow chart.

A summary of the PRISMA process may be found in Figure 1.

### Stage 4: Data characterisation and analysis

Braun and Clarke’s^58^ approach to thematic analysis was adopted to scrutinise articles with different goals and populations of medical students and doctors. It circumnavigates the context-specific and socioculturally influenced nature^42^ of medical professionalism^1-3,31,35,58-60^ and the wide range of research methodologies present among the included articles that prevent the use of statistical pooling and analysis.^58,61-66^

The members of the research team independently reviewed and ‘actively’ read the included articles to find meanings and patterns in the data to construct ‘codes’ from the ‘surface’ meaning of the text.^58,67-69^ The initial codes from ‘open coding’ were grouped into categories then into themes. The reviewers reconvened at online and face-to-face meetings to discuss their individual findings.

### Stage 5: Collating, summarising, and reporting the results

A total of 3799 abstracts were identified from four databases, 138 full-text articles reviewed, and 74 full-text articles were analysed.

The narrative produced was guided by the Best Evidence Medical Education (BEME) Collaboration guide^64^ and the STORIES (Structured approach to the Reporting In healthcare education of Evidence Synthesis) statement.^70^

In addition, two reviewers carried out individual appraisals of quantitative studies using the Medical Education Research Study Quality Instrument (MERSQI)^71^ and the Consolidated Criteria for Reporting Qualitative Studies (COREQ)^72^ to evaluate the quality of qualitative and quantitative studies included in this review. They met face-to-face to reconcile any differences in their assessments and forwarded a consensus-based appraisal of the included studies. A summary of these quality assessments may be found Online Appendix B.

### Stage 6: Consultations with expert team and key stakeholders

Whilst the findings were well-received by the expert team, several members stressed the need to consider the impact of the diverse assessment methods, approaches, goals, and populations in relation to the local educational, healthcare and linguistic setting. This is so as to facilitate development of a cohesive, longitudinal and holistic system of assessments with appropriate integration of individual assessment tools calibrated for various training stages within the Singaporean setting.

## Results

For transparency and ease of review the themes are delineated in tables. The initial themes identified were attributes and assessment criteria for professionalism (Table 3a and b); tools, approaches and modalities used to assess professionalism (Table 4); and prevailing assessment frameworks established by healthcare regulatory organisations (Table 5).

**Table 3a.** Attributes of professionalism.

General attitudes and behaviour • Confidence^73^ • Innovation^74^ • Collegiality^73^ • Altruism^21,24,29,75-77^ • Self-motivation^78,79^ • Commitment to lifelong learning^4,73,75,77,78,80^ • Commitment to profession^23,73,75,81,82^ • Upholding standards of excellence^21,29,76,83^	Ethical attitudes and behaviour • Integrity^21,24,29,30,73-77,83-87^ • Equity^24^ • Respect^21,24,25,73,76-78,85,88-91^ • Confidentiality^75,77,83,84,87,88,91,92^ • Trustworthiness^21,23,29,30,93,73,77,78,80,81,84-87,94-97^ • Responsibility^21,28,75,82,83,88^ • Accountability^77,78,81,83,85,86,88,98-100^ • Upholding justice and social responsibility^21,75,78,80,89,92,95,100^ • Upholding moral fiduciary relationship and duty^24,82^	Patient care • Patient-centred approach^78,89,90,95-97^ • Empathy^85,87,90-92,97,101^ • Humanism^73,80,82,102-104^ • Compassion^75,77,83,84,87,88,91,92^ • Cultural sensitivity and proficiency^29,73,77,80,82,91-93,97,102^

**Table 3b.** Assessment criteria for professionalism.

Attitudes	• (Perceived) facilitators and barriers to professional and unprofessional behaviour^25,85,100,105-107^ • Awareness of exposure to unprofessional behaviour in the informal and hidden curriculum^28-30,86,92,100,105,107-110^ • Adopting a humanistic attitude towards medicine^10^ • Self-efficacy in acquiring professionalism skillsets and in interpersonal interactions and teamwork^86,89,93,105,111,112^ • Focusing on patient’s needs above self and empathising with and respecting patient’s dignity^90,91,96,101,103,106^
Behaviours	Individual level • Balancing competing demands of research, clinical, personal and fellowship life^90^ • Asking for help when needed^87^ such as for mental and physical abuse^87^ • Maintaining composure in difficult situations^87,104^ • Establishing personal-professional lifestyle balance • Ensuring “protected time” for scholarly pursuits and spontaneously pursuing own area of interest^106,113^ • Adhering to and role modelling ethical conduct and professionalism^25,29,77-79,100^ • Respecting and cooperating with authority^4,73,80^ • Displaying lifelong learning and adaptability as well as taking initiatives, being punctual and attentive^4,23,26,30,73,77-80,85,87,97,104,105^ • Responding constructively to assessments and criticism^23,30,73,75,77-79,87,91,92,93,97,99,104,114^ Interpersonal level • Ensuring the following unprofessional behaviours are not performed • Acting compulsively^30^ • Venting emotions against patients, family, and colleagues^90,96^ • Using derogatory language^92,104^ • Pushing the blame onto others^112^ • Denigrating the medical profession^30^ • Respecting and collaborating with fellow healthcare professionals^30,78,90,97,104,111^ • Participating proactively in team-based learning^115^ • Teaching and assisting peers, juniors and students^78,101,113^ • Providing constructive feedback,^25,74,89,92,97,100,101,111,115^ accepting and soliciting feedback^73,74,81,86,92,104,115^ • Reporting dishonesty^78^ • Avoiding abuse of power^78,92^ • Respecting and collaborating with patients^30,78,89,90,95,96,103,104,111^ • Providing holistic care and showing interest in patient as a person^73,80,82,102-104^ • Sharing decision-making with patients and acknowledging and advocating for patient autonomy^91,92,97,103^ • Communicating honestly^92,103^ and empathetically^92,101,103,104^ • Respecting patient’s family^30,78,89,90,95,104^ • Managing conflicts of interest^78,95^ Organisational and Societal Level • Involved in citizenship and professional engagement^85^ • Treating underprivileged patients and improving access to medical care such as through joint and appropriate distribution and use of healthcare resources^78,82,89,95,104,113^ • Bringing positive influence to the work environment^87^
Clinical skills and competencies	Interpersonal skills with fellow healthcare professionals • Communication,^30,83,92,104^ leadership,^87,89,99^ teamwork,^77,89,99^ educational knowledge and competencies^94^ Interpersonal skills with patients • Communicating well with patients^25,26,73,74,80,84,85,88,89,91,92,94,96-98,104,105,112,116^ such as appropriately breaking bad news,^93,112^ and discerning appropriate language to use to ensure patient understands^85,96^ Self-development skills • Coping against depression and burnout^23,81,87,90^ • Self-directed learning,^23,78,88,97-99,104,111,113^ self-awareness and self-regulation^30,73,75,83,92,93,104^ • Reflectivity^4,104^ • Time management^104^ • Problem-solving and critical thinking^91^ Clinical skills • Risk assessment^89,99,104,112,114^ • Clinical decision-making^82,89,94,96,97,99,104,114^ and situational awareness in times of medical or ethical dilemma^89,91,96,99,104,114^ • Quality and error management^23,30,73,75,77-79,87,91-93,97,99,104,114^ Competencies in procedural medical skills,^89^ providing continuity of care,^87,89,104,114^ medication prescription and counselling,^93,96,97,114^ caring for the dying,^90^ obtaining informed consent^93^ and medical documentation^112^
Other measures	• Improvement in clinical^117^ and evidence-based practice^73^ • Improvement in patient satisfaction and reduction in complaints^118^

**Table 4.** Tools, approaches and modalities used in the assessment of professionalism.

Assessment approaches and modalities	
Tool	• Dreyfus and Dreyfus Level of Mastery^105^ • Miller’s Performance Level^3,26,74,85^ • Pangaro’s Performance Level^85^ • REFLECT rubric^119^ • Kirkpatrick model^118^ • Entrustable Professional Activities^21,23,29,74-76,79,84,87,89,93,94,96,98,105,119-121^
Setting	• Undergraduate medical school education^18,20,24-26,28,74,80,83,85,96,98,104,106,107,109,111,115,117,119,120,122,123-127^ • Graduate medical school education^88^ • Postgraduate clinical training^4,21-23,29,30,76-79,81,86,90,93-95,97,99,100,102,103,105,108,110,112-114,116,118,121,128^ • Continuing professional development from medical school education to medical doctor training^3,75,84,101,129,130^
Target population	• Medical Students^18,20,24,25,28,83,85,96,98,104,106,107,109,111,115,117,119,120,122-127^ • Residents^4,23,29,30,77,79,81,86,87,89,93,100,102,103,108,110,112,114,116,121^ • Medical Doctors^21,22,76,78,82,94,95,97,99,100,113,118,128,131^ • Clinical fellows^90^ • Faculty staff or tutors^75,92,100,101,105,123^
Methods	Mixed method • Questionnaire^22,75,83,85,88,92,96,100,109,112,114,119,120,122,124,131^ • Portfolio^4,122^ Quantitative • Likert Scale Questionnaire^22-24,28-30,76-78,83,85-88,90,92-94,96,97,99,101-107,109,110,111,112,116-122,124-127,129^ • Frequency of behaviour scale^21,84^ • Yes/No questionnaire^76,84^ • Checklist^84,115,128^ • Multiple-choice questions/responses^118,131^ Open-ended • Interview^25,81,85^ • Reflective Writing/Diary^85,100,119^ • Focus group discussion^84,124^ • Open-ended Questionnaire-Free Text and Essay^22,83,85,88,92,96,97,109,114,120,124,131^ • Comment card^122^
Goals	• Formative^21,98,117^ • Summative^93,95,104^
Source	• Multi-modal^75,118,122^ • Multi-source^3,4,23,26,30,74,80-84,105,108,115,116,122,130^ • Self-assessment^20,21,24,25,28,76-79,83,85,88-90,97-100,106,109,111-117,119-121,126,127,131^ • Peer-assessment^83,97^ • Faculty-assessment^29,86,87,93,95,100,104,107^ • Patient-assessment^85,96,97,103^ • Nurse-assessment^23,80,116^

**Table 5.** Assessment frameworks established by healthcare regulatory organisations.

Assessment frameworks
• Accreditation Council for Graduate Medical Education (United States) • American Board of Internal Medicine Medical Professionalism Framework • American Board of Medical Specialties • American College of Physician Professionalism Domains • Canadian Medical Education Directives for Specialists Roles for Family Medicine • European Federation for Internal Medicine’s Physician Charter for Medical Professionalism • General Medical Council Professionalism Capabilities Framework (United Kingdom) • Indian Medical Council Professional Conduct, Etiquette and Ethics International Classification of Primary Care Competency

Upon recommendation from the expert team, these three initial themes were reconfigured into two in this review^132-134^ – the context-specific and competency-based nature of assessments. The competency-based nature of assessments correspond to various stages of a medical student’s or doctor’s training (henceforth competency-based stages).

### Context-specific nature of professionalism assessments

Although many of the included articles adopted definitions of medical professionalism outlined by frameworks established by key healthcare regulatory organisations, their assessments were revealed to be significantly context specific. For example, whilst the ACGME definition was adopted by Hochberg et al^93^ and Fontes et al^23^ in surgical education, the former’s assessments focused on the prevailing surgical culture, the informal and hidden curriculum, and specific competencies practised in their specialty. The latter, however, focused on “exposure to professionalism and interpersonal and communication skills concepts”. In further contrast, Malakoff et al’s^108^ assessments of internal medicine and transitional year residents revealed a heavier focus on participation in events such as grand rounds and sponsored conferences despite following the same ACGME definition guidelines.

Differences in settings, specialties, target populations and rationales also influenced assessments of professionalism. Further examples of the context-specific nature of assessments of professionalism are featured in Table 6.

**Table 6.** Shared professionalism frameworks with variances in assessment context and domains.

Framework	Author, year	Setting	Rationale	Key domains assessed							
				PCAC	HACP	MEPMH	PCPPG	Commitment to:			
								Patients	Society	Profession	Self
Accreditation Council for Graduate Medical Education (ACGME)	Chandler et al^116^	Postgraduate (PG), Paediatrics, Residents	To determine if non-faculty ratings of resident professionalism and interpersonal skills differ from faculty ratings through use of 360-degree evaluations suggested by ACGME	X							
ACGME	Fontes et al^23^	PG, Neuro surgery, Residents	To foster teaching and continuous evaluation of ACGME core competencies through findings from two interventions implemented on residents	X		X	X			X	X
ACGME	Gauger et al^30^	PG, Surgery, Residents	To develop an instrument to measure specific aspects of professionalism in surgical residents	X							
ACGME, American Board of Medical Specialties (ABMS), American Board of Internal Medicine (ABIM), Association of American Colleges (AAC)	Gillespie et al^29^	PG, Various, Senior residents in emergency medicine, internal medicine, paediatrics, psychiatry and surgery	To assess perceptions of professional competence and professionalism in residents’ learning environment	X							
ACGME	Hochberg et al^93^	PG, Surgery, Residents	To assess whether professionalism has taken root in their surgical resident culture three years after implementing professionalism curriculum	X	X	X	X				
ACGME	Malakoff et al^108^	PG, Internal medicine, Medical students and transitional year residents	To objectively assess professionalism	X							
ACGME	Picho et al^79^	Undergraduate (UG)	To assess alumni perceptions of clinical practice preparedness using ACGME competencies	X							
ACGME	Rawlings et al^87^	PG, General surgery, Residents	To develop and evaluate narrative cases representing the five levels of the ACGME professionalism milestones	X							
ACGME	Santosa et al^121^	PG, Psychiatry, Residents	To develop an ACGME-adapted instrument to assess professionalism in psychiatric residents	X							
ACGME	Tanaka et al^81^	PG, Anaesthesiology, Residents	To define optimal professionalism feedback; develop, test, and implement a web-based feedback tool; and map the results to ACGME anaesthesiology milestones	X							
ACGME ABMS	Williams et al^105^	PG, Physicians at a large Midwestern regional healthcare provider and hospital system	To determine convergence of Miller’s framework with the ACGME/ABMS Core Competency framework	X							
General Medical Council (GMC)	Campbell et al^97^	PG, Non-training grade doctors, colleagues and patients	To investigate potential sources of systematic bias arising in the assessment of doctors’ professionalism	X			X	X		X	X
GMC	Johnston et al^109^	UG, Medical students at Queen’s University, Belfast, United Kingdom	To assess professional attitudes at different curriculum stages and investigate the influence of the hidden curriculum using a novel tool based on GMC standards	X							
GMC	Olsson et al^94^	PG, Family Medicine, Residents	To assess the internal consistency of components in the Swedish adaptation of the GMC questionnaires and to determine aspects of good medical practice reflected in their latent variable structure	X	X						
Canadian Medical Education Directives for Specialists (CanMEDS)	Al-Abdulrazzaq et al^19^	UG, Final year medical students at Kuwait University	To explore experiences and views of Kuwait students on professionalism					X	X	X	
CanMEDS	Kalen et al^120^	UG, Medical students at Karolinska Institutet, Sweden	To explore perceptions of specific learning activities and their relation to professional development as defined by CanMEDS			X	X				
CanMEDS	Ortwein et al^99^	PG, Anaesthesiology, Specialist and registrar	To evaluate the validity of a core competency catalogue based on CanMEDS and to ascertain differences in perceptions by specialists and registrars	X	X		X	X		X	
CanMEDS	Peterkin et al^119^	UG, Third year medical students	To determine if using reflective writing in teaching roles delineated by CanMEDS increases students’ understanding of clinical roles	X							
CanMEDS	Rademakers et al^127^	UG, Sixth year medical students	To determine value of specific CanMEDS competencies as perceived by students	X							
CanMEDS	Warren et al^75^	PG, Canadian residency program directors in anaesthesiology, diagnostic radiology, general surgery, internal medicine, obstetrics and gynaecology, paediatrics and psychiatry	To understand the program directors’ perceptions of the CanMEDS Professional Role, and identify teaching and assessment methods employed				X				
American Board of Internal Medicine (ABIM)	Askarian et al^76^	UG, Medical students	To investigate perceptions of professional behaviour exhibited by peers	X							
American Board of Internal Medicine (ABIM)	Blackall et al^24^	UG, Medical students	To evaluate the development and factorial validity of an instrument used to measure attitudes toward professionalism in medical education among students, residents and faculty	X							
ABIM American College of Physicians (ACP) European Federation of Internal Medicine (EFIM)	Humphrey et al^84^	UG, Preclinical and clinical students, and residents	To increase awareness of medical professionalism across institution and better understand changes in medical trainees’ professional behaviours as a result of learning environment	X	X	X	X				
ABIM	Roberts et al^123^	UG, Faculty	To determine unprofessional behaviours endemic in their institution and particular departments	X							
ABIM	Tsai et al^20^	UG, Seventh year medical students	To identify and understand structure of latent traits underlying concept of medical professionalism for Taiwanese students	X							
Indian Medical Council (IMC)	Bahus Førde^135^	UG, Medical students at a private medical school in Pondicherry, India	To evaluate awareness of the ethical code of conduct for medical practitioners	X							

Abbreviations: PCAC, Professional Conduct and Accountability; HACP, Humanism and Cultural Proficiency; MEPMH, Maintaining Emotional, Physical and Mental Health; PCPAPG, Pursuing Continual Personal and Professional Growth.

### Competency-based stages in professionalism assessment

The competency-based nature of assessments is evidenced by ten studies which put forth a stepwise approach to the assessment of professionalism knowledge and skills informed by the learner’s development.^23,93,100-102,114,118,119,122,131^

In the initial stages, assessments focus on evaluating the understanding of concepts of professionalism as well as their key attributes and role in medical education. Next, perceptions and attitudes towards the roles and responsibilities of a professional are assessed. The final stage culminates in the sustained development and display of key professional attitudes and behaviours over time.^23,93,100-102,114,118,119,122,131^

The presence of such progressive and longitudinal competency-based stages highlights the need for a safe and supportive environment as well as structured training processes.^125^

## Discussion

In highlighting available tools used to assess medical professionalism as well as pertinent domains to consider, this systematic scoping review successfully meets its primary and secondary research objectives. The context-specific nature of assessments is evident in the fact that prevailing tools or systems of assessments are based on regnant characterisations^23,28,29,74,75,79,84,87,89,93,94,98,104,105,119-121^ and prioritisation of specific professional attributes of medical professionalism.^21,29,75,76,78,80,83,89,92,95,100^

However, despite these contextual differences, it is equally important to acknowledge that there are consistent features to most of them. These include use of a variety of modalities, sources of assessments, and focus on knowledge, attitudes, behaviours, skills, competencies and outcomes.^3,4,23,26,30,74,75,80-84,105,108,115,116,118,122,130^

The findings further reinforce the notion that professionalism assessments should be competency-based, highlighting the need to carefully design their various stages. It is posited here that the stages of professionalism in the included articles correspond with the stages of Miller’s Pyramid (Table 7). In turn, assessments of professionalism are consistent with Kirkpatrick’s Model of Assessment (Table 8).^23,93,118,122^ Here, it is inferred that well-established milestones and competency levels are able to guide the learner’s progress as well as inform assessment processes.

**Table 7.** Miller’s pyramid (reproduced from Moore et al^136^).

Miller pyramid	Definition	Outcomes reported
Does	Demonstration of Professionalism in Everyday Clinical Practice	Likelihood of students to report physicians, nurses and fellow students^125^
		Impact of faculty development process on faculty’s humanistic teaching^92,101^
		Medical students’ professionalism,^91,111^ manifest through behaviour and communication skills^80,83,104^ • Self-assessment^83^ • Peer-assessment^83^ • Multi-source feedback^115^ • Assessed by Patients^85^
		Physicians’ professionalism^30,22,90,96,118,122^ such as through behaviour^73,86^ and/or demonstration of Core Competencies set out by ACGME^79,89,99^ • Peer-assessment^97^ This may also be carried out via evaluating the interpersonal and communication skills,^23,116^ self-control^77^ and empathy^102^
		The professionalism curriculum as a cultural change agent^93^
Shows how	Demonstration of Professionalism in a controlled setting such as in response to clinical vignettes and case scenarios	Likelihood of students to report physicians, nurses and fellow students for unprofessional behaviour^125^
		Response to case scenarios of ethical issues^18,128^ and unprofessional behaviour^131^
		Demonstration of professional behaviour using clinical vignettes^129^ and standardised patients^103^
Knows how/Understand	Being able to articulate understanding of professionalism	Faculty understanding of feedback delivery for professionalism competency pre- and post- faculty development program^100^
Knows/Knowledge	State the definition of professionalism (but not necessarily demonstrate understanding and internalisation)	Perception and attitudes of professionalism^24,25,84,88,109,117,130^ and professional behaviour^21,76,109^
		Change in attitudes towards professionalism after professionalism course^25,109,114,117,119^
		Residents’ perceptions of their own professionalism and the professionalism of their learning environment^29^

**Table 8.** Kirkpatrick’s model of assessment.

Articles	Learning					
	Level 1 (participation)	Level 2a (attitudes and perception)	Level 2b (knowledge and skills)	Level 3 (behavioural change)	Level 4a (organisation practice)	Level 4b (patient benefits)
Aggarwal and Kheriaty^125^	X	X				
Akhund et al^126^	X					
Arnold et al^106^						
Arun Babu et al^18^			X			
Asghari et al^21^		X				
Askarian et al^76^		X				
Blackall et al^24^		X				
Branch et al^101^	X	X	X	X		
Brauch et al^100^	X	X	X			
Bryan et al^83^				X		
Campbell et al^97^				X		
Chandler et al^116^				X		
Cruess et al^104^				X		
Cuesta-Briand et al^25^		X				
Davis et al^85^		X				
Delport et al^91^				X		
Domen et al^131^	X	X	X			
Dyrbye et al^28^						
Elcin et al^117^		X				
Emanuel^82^						
Emke et al^115^				X		
Fontes et al^23^	X	X	X	X		
Gauger et al^30^				X		
Gillespie et al^29^				X		
Gisondi et al^128^			X			
Goldie^3^						
Guraya et al^26^						
Haque et al^88^		X	X			
Hershberger et al^77^			X			
Hochberg et al^93^	X	X	X	X	X	
Hultman et al^118^	X	X	X	X	X	
Humphrey et al^84^		X		X		
Iramaneerat^95^						
Johnston et al^109^		X				
Kalen et al^120^	X					
Kalet et al^122^	X	X	X	X		
Katic et al^96^				X		
Kesselheim et al^90^				X		
Mak-van der Vossen et al^107^						
Malakoff et al^108^				X		
Menna et al^80^				X		
Nagler et al^22^		X		X		
O’Sullivan, Toohey^129^		X	X			
Olsson et al^94^				X		
Ortwein et al^99^				X		
Pavon et al^114^	X	X	X			
Peterkin et al^119^	X	X	X			
Peterson et al^89^				X		
Picho et al^79^				X		
Rademakers et al^127^		X				
Raee et al^111^				X		
Rawlings et al^87^						
Roberts et al^102^	X	X	X	X		
Roberts et al^123^						
Sang et al^113^				X		
Santen et al^98^						
Santosa et al^121^				X		
Stockley, Forbes^124^						
Strowd et al^78^		X				
Sullivan et al^110^						
Tanaka et al^81^						
Taylor et al^112^			X			
Todhunter et al^92^			X	X		
Tsai et al^20^		X				
van de Camp et al^73^				X		
Vora et al^103^	X	X	X	X		
Wilkinson et al^4^						
Williams et al^74^						
Williams et al^105^						
Yazdankhah et al^86^				X		

The stages of assessments also reveal the longitudinal nature of professional development and the need for longitudinal assessments. There are a number of considerations.

One, reliance upon pre-existing knowledge and skills foregrounds the need for effective assessments prior to commencement of professionalism training. Whilst it is crucial that standards and codes of conduct are consistently applied, personalised training and assessments are also paramount as learners may have different baseline abilities and often require different forms of support at different junctures to achieve their desired goals.

Two, longitudinal assessments must be sensitive not only to the personalised aspect of professional development but also the appropriate stage of their learning. Indeed, emphasis on regular assessments require acknowledgement of the learner’s prevailing contextual, linguistic and cultural sensitivity as well as local understanding of professionalism. These also underscore the importance of discerning appropriate tools to be employed at each stage, with the learner’s movement from didactic learning to independent display of practical knowledge and skills. This stage-wise consideration is important given that the data accrued at each developmental stage will guide subsequent teaching, assessments and remediation for the learner.

Three, most tools do not incorporate multisource data required to effectively appreciate the evolving, adaptive nature of a developing professional identity in an individual and their practice. As far as possible, tools should be specific to different stages of professionalism development or be sufficiently flexible to account for differences in the learner’s abilities and setting.

Four, clear and realistic milestones must be established at each competency-based stage. Remediation plans should be also made available and actively integrated to ensure that learners in need do not slip between the cracks and are offered targeted and timely support.

Five, given that assessments are largely dependent upon the supervisor, mentor, coach and/or tutor, assessors must be trained on which tool to use and how each tool is to be effectively used. To facilitate communication between assessors in different settings and acknowledging the longitudinal nature of professional development, a portfolio-based assessment method should be employed to streamline the assessment process.

Consequently, these aspects of assessing professionalism affirms the fact that medical professionalism cannot be restricted to a *“standardised and reductionist”* definition and assessment method.^2,31,137^ Rather, these findings underline the need for a portfolio-based assessment program^4,122^ where a mixture of generalised tools and context-specific ‘specialised’ assessment methods can be employed to assess competencies and milestones achieved. The portfolio must also consider the prevailing practice culture, availability of resources and receptivity of its implementation to ensure the sustainability of professionalism assessment.

A portfolio approach would also allow reflections on individual experiences and allow assessors to evaluate and provide feedback on these reviews of experiences and refinements in practice and thinking. An additional consideration is that learners can also provide their perspective of the assessments and may even provide their views of a particular assessment and challenge the appraisal. This introduces the need for assessments to overseen and reviewed by independent third parties who may offer a review of the overall progress of the learner or a review of a particular assessment.

**Figure 2.**
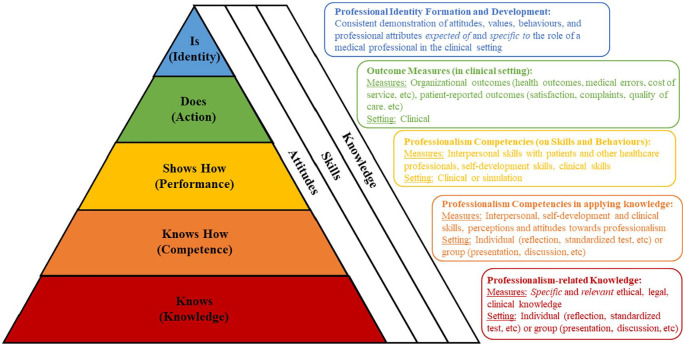
Competency-based stages of professionalism assessment.

Mapping these findings have generated much insight and a general guidance for the design of professionalism assessments is featured in Figure 2 where key principles corresponding to each competency-based stage is foregrounded.

## Limitations

Whilst this review focused solely on medical professionalism assessments, the articles included explored broader concepts of professionalism. The limited scope of this review also saw the exclusion of closely associated concepts such as professional identity formation and ethics^31^ which may have hampered a holistic picture of assessment practices in medical professionalism.^2,31,137^ Due to time, manpower and resource constraint, the exclusion of other health professional literature may have also led to the omission of key ideas potentially transferable to the field of medical education.

Moreover, as the included articles were restricted to those in English, majority of the accounts originated from North America and the European countries. Although the utilisation of similar internationally acclaimed and accredited professionalism frameworks may suggest universal similarities and agreement over the domains of medical professionalism, it is evident that clinical and educational committees evaluate and adapt their guidelines in tandem with local healthcare and educational contexts and cultures. As the findings may not be representative of professionalism assessment practices beyond these countries, this review is limited in guiding educators on assessment attributes and criteria that will overcome nuanced geographical and cultural boundaries.^1-3,31,35,58-60^

## Future directions to consider

Mapping of prevailing medical professionalism assessment practices highlight the following potential areas for future research and action:

- Delineate a working definition of medical professionalism that acknowledges their longitudinal and competency-based nature and integrates local sociocultural and contextual factors
- Establish consensus on a medical professionalism framework that incorporates universally agreeable codes of conduct whilst granting flexibility for geographical and cultural considerations and specialties
- Develop principles to guide the design of professionalism assessments with more robust understanding of how various assessment methods, criteria, content, goals, and needs may be integrated with due consideration for prevailing resources, settings and target populations
- Evaluate the significance of sociocultural and linguistic idiosyncrasies in the design of assessment tools
- Carry out more detailed and context-specific systematic reviews to better determine unique factors influencing how professionalism could be best assessed in psychiatry and other medical and surgical specialities
- Adequately train and support assessors to ensure efficacious use of assessment tools and transparently convey to learners on the specifics of what they will be assessed on and how they will be assessed
- Consider the use of portfolios which will allow for longitudinal, multidimensional reviews and learner initiated commentaries and reflections – this would provide greater opportunities for personalised remediation and training, as well as more streamlined communications between assessors

## Supplemental Material

Appendix_A_xyz4525182e020e7 – Supplemental material for Assessing Professionalism in Medicine – A Scoping Review of Assessment Tools from 1990 to 2018 Click here for additional data file. Supplemental material, Appendix_A_xyz4525182e020e7 for Assessing Professionalism in Medicine – A Scoping Review of Assessment Tools from 1990 to 2018 by Kuang Teck Tay, Shea Ng, Jia Min Hee, Elisha Wan Ying Chia, Divya Vythilingam, Yun Ting Ong, Min Chiam, Annelissa Mien Chew Chin, Warren Fong, Limin Wijaya, Ying Pin Toh, Stephen Mason and Lalit Kumar Radha Krishna in Journal of Medical Education and Curricular Development

Appendix_B_xyz4525142640e2f – Supplemental material for Assessing Professionalism in Medicine – A Scoping Review of Assessment Tools from 1990 to 2018 Click here for additional data file. Supplemental material, Appendix_B_xyz4525142640e2f for Assessing Professionalism in Medicine – A Scoping Review of Assessment Tools from 1990 to 2018 by Kuang Teck Tay, Shea Ng, Jia Min Hee, Elisha Wan Ying Chia, Divya Vythilingam, Yun Ting Ong, Min Chiam, Annelissa Mien Chew Chin, Warren Fong, Limin Wijaya, Ying Pin Toh, Stephen Mason and Lalit Kumar Radha Krishna in Journal of Medical Education and Curricular Development
