# Supplementary material for: Assessing Professionalism in Medicine – A Scoping Review of Assessment Tools from 1990 to 2018
Source: J Med Educ Curric Dev. 2020 Oct 16;7:2382120520955159. doi: 10.1177/2382120520955159 (PMC7580192; doi:10.1177/2382120520955159)
Supplement: Appendix_A_xyz4525182e020e7 – Supplemental material for Assessing Professionalism in Medicine – A Scoping Review of Assessment Tools from 1990 to 2018 [file Appendix_A_xyz4525182e020e7.pdf]

Appendix A – Search Strategy

**Search Strategy Approach:**<br>Professionalism AND Doctors/Medical Student AND Assessment AND tools AND (ACGME OR GMC OR CanMEDS)		
**Concept**	**Subject Headings (MeSH)**	**Keywords [Title/Abstract]**
Professionalism	"Professionalism"[MeSH] OR "Professional Competence"[MeSH] OR "Professional Role" [MeSH] OR "ethics, medical"[MeSH] OR "patient-centered care"[MeSH]	Professional[Title/Abstract] OR Professionalism[Title/Abstract]
Doctors/Medical Students	Physicians[MeSH] OR "Students, Medical"[MeSH] OR "Education, Professional"[MeSH] OR "Clinical Clerkship"[MeSH]	Physician[Title/Abstract] OR Physicians [Title/Abstract] OR resident[Title/Abstract] OR residents[Title/Abstract] OR residency[Title/Abstract] OR residencies[Title/Abstract] OR practice[Title/Abstract] OR practitioner[Title/Abstract] OR practitioners[Title/Abstract] OR doctor[Title/Abstract] OR doctors[Title/Abstract] OR houseman[Title/Abstract] OR housemanship[Title/Abstract] OR housemen[Title/Abstract] OR "medical officer"[Title/Abstract] OR "medical officers"[Title/Abstract] OR "medical student"[Title/Abstract] OR "medical students"[Title/Abstract]
Assessment		Assess[Title/Abstract] OR Assessment[Title/Abstract] OR Feedback[Title/Abstract] OR questionnaire[Title/Abstract] OR evaluation[Title/Abstract] OR "Multisource feedback"[Title/Abstract]
Tools		Tool[Title/Abstract] OR tools[Title/Abstract] OR Instrument[Title/Abstract] OR Rubric[Title/Abstract] OR Rubrics[Title/Abstract] Questionnaire[Title/Abstract] OR questionnaires [Title/Abstract] OR survey[Title/Abstract] OR surveys[Title/Abstract]
ACGME Professionalism Core Competency https://knowledgeplus.nejm.org/blog/acgme-core-competencies-professionalism/		"Accreditation Council for Graduate Medical Education"[Title/Abstract] OR ACGME[Title/Abstract] OR "Professional Conduct"[Title/Abstract] OR Accountability[Title/Abstract] OR Humanism[Title/Abstract] OR "Cultural Proficiency"[Title/Abstract] OR "Emotional Health"[Title/Abstract] OR "Physical Health"[Title/Abstract] OR "Mental Health"[Title/Abstract] OR "Personal Growth"[Title/Abstract] OR "Professional Growth"[Title/Abstract]
General Medical Council (GMC) Generic Professional Capabilities Framework-Professional values and behaviours		"General Medical Council Generic Professional Capabilities Framework"[Title/Abstract] OR GMC[Title/Abstract] OR "Professional Value*"[Title/Abstract] OR "Professional Behavio*"[Title/Abstract]
CanMEDS Physician Competency Framework-Professional role		"Canadian Medical Education Directions for Specialists"[Title/Abstract] OR CanMEDS[Title/Abstract] OR Commitment[Title/Abstract]
