# Supplementary material for: Assessing Professionalism in Medicine – A Scoping Review of Assessment Tools from 1990 to 2018
Source: J Med Educ Curric Dev. 2020 Oct 16;7:2382120520955159. doi: 10.1177/2382120520955159 (PMC7580192; doi:10.1177/2382120520955159)
Supplement: Appendix_B_xyz4525142640e2f – Supplemental material for Assessing Professionalism in Medicine – A Scoping Review of Assessment Tools from 1990 to 2018 [file Appendix_B_xyz4525142640e2f.pdf]

Appendix B – Summary of Included Articles

No	Author, Year	Objectives of Study	Methodology (in detail from abstract)	Assessor Population	Population of those Assessed	MERSQI	COREQ
1	Aggarwal, Kheriaty [127]	To assess the likelihood of medical students to directly approach and to report various providers—a physician, nurse, or medical student—for three behaviors (poor hand hygiene, intoxication, or disrespect of patients).	A survey was administered to 159 medical students to assess their likelihood to directly approach and to report various providers—a physician, nurse, or medical student—for three behaviors (poor hand hygiene, intoxication, or disrespect of patients).	Medical Educators from University of California, Irvine School of Medicine	Medical students	7/18.	NA
2	Akhund, et al. [128]	The primary aim of this study was to assess attitudes of Pakistani and Pakistani heritage students at a medical college in Pakistan about important elements of professionalism that an ideal medical doctor should possess. A further objective of the study was to determine students' preferred ways of learning professionalism	A written survey was distributed to undergraduate medical students at a public sector medical college at Karachi, Pakistan in 2011. Using the Penn State College of Medicine (PSCOM) Professionalism Questionnaire, attitudes of medical students of semester 1, 5, and 8 regarding professionalism were assessed anonymously	Department of Community Medicine, Dow International Medical College, Dow University of Health Sciences, Karachi, Pakistan	Undergraduate medical students at a public sector medical college in Karachi, Pakistan in the year 2011.	8/18.	NA
3	Al-Abdulrazzaq, et al. [19]	Our aim was to explore the experiences and views of Kuwait final-year medical students on professionalism.	This was a questionnaire study of final-year medical students at Kuwait University (n = 95). Open- and close-ended questions were used to determine the students' experiences and views on: definition, teaching, learning, and assessment of professionalism.	This was a questionnaire study carried out among the final-year students in the 2012–2013 academic year. The questionnaire was designed by the authors based on similar studies on medical professionalism among undergraduate and postgraduate students and residents	Final-year medical students at Kuwait University (n = 95)	7/18.	20/32.
4	Arnold, et al. [112]	This report extends previous research through a multi-institutional study of students' perspectives about system characteristics for peer assessment of professionalism. It examines whether students from different schools and year levels prefer different characteristics of peer assessment to assess each other candidly, or whether a single system can be designed. It then identifies the characteristics of the resulting preferred system(s).	At the beginning of academic year 2004-2005, students (1,661 of 2,115; 78%) in years one through four at four schools replied to a survey about which peer assessment characteristics - related to, for example, who receives the assessment, its anonymity, and timing - would prevent or encourage their participation. Multivariate analysis of variance was used to detect differences among institutions and students from each year level.	Medical Educator from Office of Medical Education and Research, University of Missouri-Kansas City School of Medicine, Kansas City, USA	The study sites for this research were four U.S. medical schools. Two are private schools in the northeast region of the United States, as classified by the Association of American Medical Colleges (AAMC).	NA	18/32.

5	Asghari, et al. [21]	This study was conducted to assess interns' level of exposure to different aspects of professional attitude and behaviour among faculty and residents.	Cross-sectional descriptive study	Medical Educators from Medical Ethics and History of Medicine Research Center, Tehran University of Medical Sciences, Tehran, Iran 2 Faculty of Medicine, Tehran University of Medical Sciences, Tehran, Iran	218 medical interns in their surgery and internal medicine rotations in four teaching hospitals affiliated to Tehran University of Medical Sciences	10.5/18.	NA
6	Askarian, et al. [81]	This study aimed to investigate the medical students' perceptions of their colleagues' professional behavior.	This study is a cross-sectional study with 280 medical students at Shiraz University of Medical Sciences in their fifth to seventh year of study as the sample. The study was performed during one month in 2013, using stratified random sampling method. The instrument of the study was the Persian version of the questionnaire of the American Board of Internal Medicine (ABIM).The questionnaire includes demographic information, questions about the meaning of the professionalism, history of medical ethics education programs and 12 behavioral questions. The data were analyzed using student t-test and Pearson correlation test. The significance level was set as 0.05.	Medical Educators from Shiraz University of Medical Sciences, Iran	Medical Students	10/18.	NA
7	Arun Babu, et al. [18]	The purpose of this study was to evaluate the awareness of the 'ethical code of conduct for medical practitioners' among medical undergraduate students	Cross-sectional study	Medical Educators from Department of Pediatrics, Sri Lakshmi Narayana Institute of Medical Sciences	172 medical students in a private medical school in Pondicherry, located in southern India.	NA	14/32.
8	Blackall, et al. [24]	The paper will describe the development and factorial validity of an instrument to measure attitudes toward professionalism in medical education among students, residents and faculty.	A factor analysis of the intercorrelations of responses to 36 items reflecting the American Board of Internal Medicine (ABIM) elements of professionalism for a sample of 765 medical students, residents and faculty was carried out. Data were collected during the spring of 2004. The study was conducted at the Penn State College of Medicine in Hershey, PA, USA.	Medical Educators from Penn State College of Medicine	Medical students	11.5/18.	NA
9	Branch, et al. [105]	To successfully design and implement longitudinal faculty development programs at five medical schools, and to determine whether faculty participants were perceived to be more effective humanistic teachers.	Twenty-nine participants who completed 18 months of faculty development at the five medical schools were compared with 47 controls drawn from the same schools in the final six months of the two-year project. For comparison, the authors developed a 10-item questionnaire, the Humanistic Teaching Practices Effectiveness Questionnaire (HTPE), to be filled out by medical students and residents taught by participants or	Expert facilitators for the faculty development groups at five medical schools (Emory University School of Medicine, Indiana University School of Medicine, the	Medical students and residents taught by participants or control faculty at five medical schools. Program participants were	14/18.	NA

			control faculty. Items were designed to measure previously identified themes and domains of humanism. Control faculty were similar to participants by gender, specialty, and years of experience.	University of Rochester School of Medicine, Baylor College of Medicine, and the University of Minnesota Medical School) were drawn from a group that has engaged in studying and teaching the human dimensions of care. The facilitators, one of whom was the principal investigator (W.T.B.) for the present study, were also designated as site leaders, responsible for implementation and partial design of the curriculum, selection of participants, and organization of the faculty development programs at their respective institutions.	selected by the facilitators (site leaders) at each of the schools. Selection was open to faculty from all departments. All served as teaching attendings on inpatient services or preceptors in teaching clinics. An effort was made within and across sites to achieve diversity by age and gender. In addition to being willing to participate, the main criterion used for selecting participants was that the individuals were considered promising as teachers and role models in the clinical setting. The faculty development group at each school included at least 8 but no more than 12 participants. Occasionally, chief residents were chosen, but they were not included in the evaluation.		

10	Brauch, et al. [104]	1) To determine the level of faculty (n=8) understanding and comfort in providing feedback, 2) the objectives of this qualitative assessment were to evaluate faculty understanding of the professionalism competency and their comfort with feedback delivery on the competency, to provide faculty development based on the preliminary assessment, and to evaluate understanding and comfort following the faculty development	Surveys listing 12- month professionalism milestones were distributed to core internal medicine teaching faculty. . Current interns (n¼10) also rated their understanding of the same milestones. The faculty development program included interpersonal communication education, role-plays of difficult situations, and pocket resources, as well as direct feedback on videotaped sessions with residents. At the end of the intervention period, participating faculty completed a postdevelopment survey, and the current 6-month interns completed a follow-up assessment.	Medical Educators from Department of Internal Medicine, Akron General Medical Center, Ohio	Postgraduate year 1 residents (interns)	7/18.	14/32.
11	Bryan, et al. [88]	The goal of this study was to determine if peer evaluation and self-evaluation used in conjunction and implemented early in the medical curriculum, can serve as useful tools to assess and provide feedback regarding professional behaviour in first-year medical students.	From 1999 to 2003, students at Mayo Clinic College of Medicine evaluated themselves and their peers during the Gross and Developmental Anatomy Course. Numerical evaluations and written comments were statistically analysed within established categories of professionalism and correlated with academic performance, gender, and peer rating and self-rating.	Medical Educator from Department of Anatomy, Division of Gastroenterology and Hepatology, Mayo Clinic College of Medicine	From 1999 to 2003, five consecutive classes at the Mayo Clinic College of Medicine (n = 213 students) were asked to evaluate professionalism in themselves and their colleagues during the first-year Gross and Developmental Anatomy Course	10/18.	14/32.
12	Byram [27]	This study employed a longitudinal approach to PIF that explored the processes through which professional identity is formed in second (MS2) and third (MS3) year medical students and how their perceptions of professionalism transformed and influenced their PIF.	Nine medical students (n=9) from Indiana University School of Medicine completed this study spanning MS2 and MS3. Participants completed three semi-structured interviews and submitted 10 audio diaries at two-month intervals between interviews. Participants also completed the Professionalism Assessment Tool (PAT) at the beginning of MS2 (PAT1) and end of MS3 (PAT2). Interviews and audio diaries were analyzed using the constant comparative approach and a Wilcoxon signed-rank test was used to determine significant differences between mean domain scores of PAT1 and PAT2.	Medical Educators from Indiana University	Second and Third Year Medical Students	15.5/18.	27/32.
13	Campbell, et al. [101]	To investigate potential sources of systematic bias arising in the assessment of doctors' professionalism.	Linear regression modelling of cross sectional questionnaire survey data	NA	1065 non-training grade doctors from various clinical specialties and settings, 17 031 of their colleagues,	11/18.	NA

					and 30 333 of their patients.		
14	Chandler, et al. [120]	The objective of our study was to use ‘‘360-degree evaluations,’’ as suggested by the Accreditation Council for Graduate Medical Education (ACGME), to determine if nonfaculty ratings of resident professionalism and interpersonal skills differ from faculty ratings.	Pediatrics residents were enrolled in a hospitalbased resident continuity clinic during a 5-week period. Patient/families (P/Fs), faculty (MD [doctor of medicine]), nurses (RNs [registered nurses]), and residents themselves (self) completed evaluator-specific evaluations after each clinic session by using a validated 10-item questionnaire with a 5-point Likert scale. The average Likert score was tallied for each questionnaire. Mean Likert scale scores for each type of rater were compared by using analysis of variance, text with pair-wise comparisons when appropriate. Agreement between rater types was measured by using the Pearson correlation.	Nurse, Faculty, Patient/Family, and Resident Self-evaluation	Paediatric Resident Physicians	12/18.	NA
15	Cruess, et al. [108]	As the evaluation of professional behaviors has been identified as an area for development, the Professionalism Mini-Evaluation Exercise (P-MEX) was developed using the mini-Clinical Examination Exercise (mini-CEX) format.	From a set of 142 observable behaviors reflective of professionalism identified at a McGill workshop, 24 were converted into an evaluation instrument modeled on the mini-CEX. This instrument, designed for use in multiple settings, was tested on clinical clerks in medicine, surgery, obstetrics and gynecology, psychiatry, and pediatrics. In all, 211 forms were completed on 74 students by 47 evaluators.	Instructions on using the form were given to all evaluators, who determined when an activity would be evaluated.	Testing proceeded on students during third- and fourth-year clerkships in Internal Medicine, General Surgery, Pediatrics, Psychiatry, and Obstetrics and Gynecology at McGill University.	15/18.	NA
16	Cuesta-Briand, et al. [25]	This paper presents results from a study exploring medical students' views on professionalism, and reports on students' constructs of the ‘good’ and the ‘professional’ doctor.	1) Data for this qualitative study were collected through focus groups conducted with medical students from one Western Australian university over a period of four years. Students were recruited through unit coordinators and invited to participate in a focus group. De-identified socio-demographic data were obtained through a brief questionnaire. Focus groups were audio-recorded, transcribed and subjected to inductive thematic analysis. 2) Differences between students' understandings of the ‘good’ and ‘professional’ doctor were observed. Being competent, a good communicator and a good teacher were the main characteristics of the ‘good’ doctor. Professionalism was strongly associated with the adoption of a professional persona; following a code of practice and professional guidelines, and treating others	Focus groups were run by expert facilitators who were neither medical educators nor connected with the medical school.	medical students from one Western Australian university over a period of four years. A total of 49 medical students took part in 13 focus groups. 2) The students recruited for this study were in the clinical years (fourth to sixth year) of The	NA	17/32.

			with respect were also associated with the 'professional' doctor.		University of Western Australia (UWA) MBBS programme.		
17	Davis, et al. [90]	Evaluate medical students' communication and professionalism skills from the perspective of the ambulatory patient and later compare these skills in their first year of residency.	Prospective Study	y as approved by theVA research committee and the University of New Mexico Human Research Review Committee.	This prospective study ran from March 2007 to July 2009 in the general neurology clinics at the New Mexico Veterans Affairs (VA) Health Care System. Students at the University of New Mexico School of Medicine spend part of their clerkship year training in VA general neurology clinics and were eligible to participate in the study a	11/18.	10/32.
18	Delport, et al. [96]	To document the development of the Charter for Medical Professionalism and to evaluate lecturer and student perceptions on the formulation of the Charter to make appropriate changes and increase acceptance.	An online survey was conducted to assess lecturer and student acceptance of the Charter.	working group comprising academics from UP's Faculty of Health Sciences developed the Charter from relevant source documents, employing thematic and content analysis and recursive abstraction A representative working group was comprised from the School of Medicine, UP, and included a health sciences education advisor, family physician-lecturer, Department of Psychiatry lecturer, and	All lecturers from the School of Medicine were invited via corporate email to participate in the study. Following incorporation of comments from lecturers, students were invited to participate anonymously in the survey via a pop-up message and link on the university's learning management system.		16/32.

				the Procedural Skills Unit head.			
19	Domen, et al. [133]	This article presents the survey results as well as basic considerations for program directors and department chairs to consider when confronted with unethical or unprofessional behavior in their resident trainees.	. We used a case-based educational approach in a workshop setting to assist program directors in the management of unprofessional behavior in residents. Eight case scenarios highlighting various aspects of unprofessional behavior by pathology residents were developed and presented in an open workshop forum at the annual pathology program director's meeting. Prior to the workshop, 2 surveys were conducted: (1) to collect data on program directors' experience with identifying, assessing, and managing unprofessional behavior in their residents and (2) to get feedback from workshop registrants on how they would manage each of the 8 case scenarios.	Graduate Medical Education Committee (GMEC) of the College of American Pathologists (CAP)	all program directors on the PRODS listserv in April 2014 program directors who had preregistered for the workshop as of June 11, 2014	NA	16/32.
20	Dyrbye, et al. [28]	To determine the relationship between measures of professionalism and burnout among US medical students.	Cross-sectional survey of all medical students attending 7 US medical schools (overall response rate, 2682/4400 [61%]) in the spring of 2009. The survey included the Maslach Burnout Inventory (MBI), the PRIME–MD depression screening instrument, and the SF-8 quality of life (QOL) assessment tool, as well as items exploring students' personal engagement in unprofessional conduct, understanding of appropriate relationships with industry, and attitudes regarding physicians' responsibility to society.	Medical Educators of 7 Medical Schools in United States (Mayo Medical School, University of Washington School of Medicine, University of Chicago Pritzker School of Medicine, University of Minnesota Medical School, University of Alabama School of Medicine, University of California-San Diego School of Medicine, and the Uniformed Services University of the Health Sciences)	Medical students	11.5/18.	NA
21	Elcin, et al. [121]	Students start the course called ''Health-Illness Concepts and Medical Professional Identity''. Students are expected to discuss the subjects given to them as scenarios or arguments. The aim of our study is to determine whether the course leads to a positive change in students' attitudes, indicating the attainment of those attributes that reflect professionalism.	1) We welcome our new students with an openning ceremony on their first day and two months later, the students start the course called ''Health-Illness Concepts and Medical Professional Identity''. Students are expected to discuss the subjects given to them as scenarios or arguments. 2) We had 22 groups, consisting of 12 students each. Eighteen faculty members took part as tutors. On the first and last days of the course, a questionnaire was delivered to the students (Figure 1). There were seven statements in the questionnaire, each focusing on a different attribute of professional behaviour. 3) Any positive change in preferences	Hacettepe University Faculty of Medicine	Hacettepe University First Year Medical Students	10/18.	NA

			between the two questionnaires is identified as an achievement of that attribute				
22	Emanuel [87]	1) Forceful new demands for accountability in medicine are arising from many interested parties. To maintain professional standards, physicians need to establish which demands are desirable and which are not. 2) Our goal is to offer, by way of a test case, tangible recommendations for accountability. We focus on a domain that is often ignored in discussions of accountability, namely, the ethical dimensions of medical care—practices, such as informed consent, that are distinct from diagnostic and procedural skills. Of all the possible domains of accountability, we concentrate on ethics because it is among the most challenging to evaluate and because it is crucial if the medical profession is to maintain the trust of patients and the public	1) We first identify eight widely endorsed content areas for accountability in ethical conduct: medical decision making, confidentiality, fiduciary obligations (including conflicts of interest), responsibilities arising from patient vulnerability, personal standards, equity among patients, cultural representation, and procedures for resolving dilemmas. We then identify the currently most valid and reliable methods for assessing conduct: surveys among all involved parties, testing methods used for accreditation, limited audits, publication of policy, and careful use of report cards. A prototypical survey and report card are illustrated. However, we also note the need for improved accountability assessment methods. We next identify mechanisms for taking responsibility: sharing information, exchanging perspectives, making adjustments, and enforcing standards when necessary. 2) We then identify the currently most valid and reliable methods for assessing conduct: surveys among all involved parties, testing methods used for accreditation, limited audits, publication of policy, and careful use of report cards.	Working Group on Accountability convened to identify the norms of ethical medical practice and to delineate desirable methods of assessment. It is an ad hoc working group of clinician ethicists; most members and consultants are practicing clinicians, and each has a specialty focus in professional ethics, medical assessment, organization, or policy	To begin describing the model, we focus on physicians and health care institutions. We focus on the ethical dimensions of medical practice, both because the difficulty of measuring such behaviors makes this a test case for accountability and because of the importance of ethical standards in maintaining patient trust.	NA	10/32.
23	Emke, et al. [119]	1) Most professionalismassessments in medical training are delayed until clinical rotations where multisource feedback isavailable. This leaves a gap in student assessment portfolios and potentially delays professionaldevelopment. 2) This study seeks to demon-strate that perceptual errors related to professionalismbehaviors can be detected early in medical trainingthrough repeated instances of context-specific multi-source feedback, creating the conditions necessary forearly, targeted remediation.	A total of 246 second-year medical students (2013–2015) completed self-and peer assessments of professional behaviors in 2 courses following a series of Team-BasedLearning exercises. Correlation and regression analyses were used to examine the alignment ormisalignment in the relationship between the 2 types of assessments. Four subgroups were formedbased on observed patterns of initial self- and peer assessment alignment or misalignment, andsubgroup membership stability over time was asses	This study was reviewed by the Washington UniversityInstitutional Review Board	A total of 246 second-year medical students (2013–2015	12.5/18.	NA
24	Fontes, et al. [23]	Incorporation of the 6 ACGME core competencies into surgical training has proven a considerable challenge particularly for the two primarily behavioral competencies, professionalism and interpersonal and communication skills. We report on experience with two specific interventions to foster the teaching and continuous evaluation of these competencies for neurosurgery residents	In 2010, the Society of Neurological Surgeons (SNS) organized the first comprehensive Neurosurgery Boot Camp courses, held at six locations throughout the US and designed to assess and teach not only psychomotor skills but also components of all six Accreditation Council for Graduate Medical Education (ACGME) core competencies. These courses are comprised of various educational methodologies, including online material, faculty lectures, clinical scenario and group discussions, manual skills stations, and pre- and post-course assessments. Resident progress in each of the 6 ACGME competencies is now tracked using the neurosurgical	Society of Neurological Surgeons (SNS)	Surgical Residents	14/18.	NA

			Milestones, developed by the ACGME in collaboration with the SNS. In addition, the Milestones drafting group for neurosurgery has formulated a milestone-compatible evaluation system to directly populate Milestone reports. These evaluations utilize formative, summative, and 360-degree evaluations that are considered by a faculty core competency committee in finalizing milestones levels for each resident.				
25	Gauger, et al. [30]	No universally accepted method to measure professionalism exists. We developed an instrument to measure specific aspects of professionalism in surgical residents.	Professionalism was deconstructed into 15 domains. Behavioral descriptors were determined for extreme and selected intermediate anchors. It became evident that residents could "go too far" in some professional behaviors. Therefore, although a 7-point continuous ordinal scale forms the framework, a score of 7 does not necessarily indicate the ideal. This characteristic minimizes the problem of inflated ratings.	Medical Educator from Department of Surgery, Division of Endocrine Surgery, Medical Center Dr., Taubman Center, USA	Surgical Residents	13/18.	NA
26	Gillespie, et al. [29]	The competency of professionalism encompasses a range of behaviors in multiple domains. Residency programs are struggling to integrate and effectively assess professionalism.We report results from a survey assessing residents' perceptions of their professional competence and the professionalism of their learning environment.	A survey was developed to assess specific behaviors reflecting professionalism based on the conceptualizations of key accrediting bodies. Residents rated their ability to perform the behaviors and reported the frequency with which they observed their fellow residents failing to perform the behaviors. Eighty-five senior residents in emergency medicine, internal medicine, pediatrics, psychiatry, and surgery specialties completed the survey (response rate577%). Differences among domains (and among items within domains) were assessed. Correlations between perceived professionalism and the professionalism of the learning environment were described.	Medical Educators from New York University School of Medicine	Eighty-five senior residents in emergency medicine, internal medicine, pediatrics, psychiatry, and surgery specialties	11/18.	NA
27	Gisondi, et al. [130]	To examine the responses of emergency medicine residents (EMRs) to ethical dilemmas in high-fidelity patient simulations as a means of assessing resident professionalism.	This cross-sectional observational study included all EMRs at a three-year training program. Subjects were excluded if they were unable or unwilling to participate. Each resident subject participated in a simulated critical patient encounter during an Emergency Medicine Crisis Resource Management course. An ethical dilemma was introduced before the end of each simulated encounter. Resident responses to that dilemma were compared with a professional performance checklist evaluation. Multiresponse permutation procedure analysis was used to compare performance measures between resident classes, with the a priori hypothesis that mean performance should increase as experience increases.	This project was conducted by Medical Educators in the Simulation Center at the Veterans Administration Palo Alto Health Care System, a teaching affiliate of the Stanford University School of Medicine. Stanford University is a large, suburban, academic medical center with an annual emergency department (ED) volume of	Subjects included EMRs from the Stanford- Kaiser Emergency Medicine Residency, a three-year training program. In conjunction with the regularly scheduled yearly simulation curriculum, postgraduate year 3 (PGY-3) residents were observed at 32	12.5/18.	NA

				approximately 40,000 patients.	months of training; PGY-2 residents at 20 months of training; and PGY-1 residents during their intern orientation.		
28	Goldie [3]	The assessment of professionalism is an evolving field. This review aims to consolidate current thinking.	NA	John Goldie, General Practice & Primary Care, University of Glasgow	NA	NA	NA
29	Guraya, et al. [26]	This review aims to describe some of the popular assessment tools that are being used to assess professionalism with a view to formulate a framework of assessment of professionalism in medicine.	In December 2015, the online research databases of MEDLINE, the Educational Resources Information Center (ERIC), Elton Bryson Stephens Company (EBSCO), SCOPUS, OVID and PsychINFO were searched for full-text English language articles published during 2000 to 2015. MeSH terms “professionalism” AND “duty” AND “assessment” OR “professionalism behavioural” AND “professionalism–cognitive” were used.	NA	NA	NA	NA
30	Haque, et al. [93]	The purpose of this study is to explore professionalism in terms of its fundamental elements among medical students of Universiti Sultan Zainal Abidin (UniSZA).	This was a cross-sectional study carried out on medical students of UniSZA. The study population included preclinical and clinical medical students of UniSZA from Year I to Year V of academic session 2014/2015. The simple random sampling technique was used to select the sample. Data were collected using a validated instrument. The data were then compiled and analyzed using SPSS Version 21.	Medical Educators from Universiti Sultan Zainal	Preclinical and clinical medical students of UniSZA from Year I to Year V of academic session 2014/2015, Medical students of Universiti Sultan Zainal Abidin (UniSZA)	11/18.	NA
31	Hershberger, et al. [82]	To investigate the extent to which self-control is a component of resident professionalism, rated both by residents and their program directors.	366 residents in 13 residency programs were invited to participate in a survey study of professionalism. Participating residents completed the Professionalism – Documentation of Competence (ProDOC) (a 15-item measure of professionalism developed for the study), a 10-item version of the Marlowe–Crowne Social Desirability Scale, and the Brief Self-Control Scale. Independently, program directors of participating residents completed the ProDOC with reference to each of their participating residents.	Medical Educators from Wright State University Boonshoft School of Medicine	Residents	13.5/18.	NA

32	Hochberg, et al. [60]	The goal of this study was to assess whether professionalism has taken root in the surgical resident culture 3 years after implementing our professionalism curriculum.	Evidence was derived from 3 studies: (1) annual self-assessments of the residents' perceived professionalism abilities to perform 20 defined tasks representing core Accrediting Council on Graduate Medical Education professionalism domains, (2) objective metrics of their demonstrated professionalism skills as rated by standardized patients annually using the objective structure clinical examination tool, and (3) a national survey of the Surgical Professionalism and Interpersonal Communications Education Study Group.	Medical Educators from New York University Medical Center	Surgical Residents	13.5/18.	NA
33	Hultman, et al. [61]	The purpose of this project was to evaluate the effectiveness of a professionalism curriculum in an academic plastic surgery practice.	We created and conducted a 6-wk, 12-h course for health care professionals in plastic surgery (faculty, residents, nurses, medical students). Teaching methods included didactic lectures, journal club, small group discussions, and book review. Topics included: (1) Professionalism in Our Culture, (2) Leadership Styles, (3) Modeling Professional Behavior, (4) Leading Your Team, (5) Managing Oneself, and (6) Leading While You Work. Using Kirkpatrick methodology to assess perception of the course (level 1 data), learning of the material (level 2 data), effect on behavior (level 3 data), and impact on the organization (level 4 data), we compiled participant questionnaires, scores from pre- and post-tests, and such metrics as incidence of sentinel events (defined as infractions requiring involvement by senior administrators), number of patient complaints reported to Patient Relations, and patient satisfaction (Press Ganey surveys), for the 6 mo before and after the course.	Medical Educators from University of North Carolina	Health care professionals in plastic surgery (faculty, residents, nurses, medical students).	15/18.	NA
34	Humphrey, et al. [89]	The authors describe the institution's approach and progress to date (regarding an institution-wide Roadmap to Professionalism designed to both increase awareness about issues of medical professionalism across the institution and gain a better understanding of how medical trainees' professional behaviors' change during their training as a result of the medical learning environment.)	The authors describe the institution's approach and progress to date. To gain buy-in from all levels of learners at the Pritzker School of Medicine, the initiative uses vertically integrated advisory groups to engage medical trainees in the assessment and development of experiential workshops and evaluation initiatives based on the principles outlined in the American Board of Internal Medicine / American College of Physicians / European Federation of Internal Medicine's Physician Charter for Medical Professionalism. Advisory groups provide targeted assessments and programming at each stage of the medical learner: preclinical students, clinical students, residents, and faculty. Surveys of medical students' perceptions of professionalism have provided an important baseline assessment of the learning environment, from which the professionalism steering committee plans to target future curricular interventions. Efforts to engage residents have focused on experiential workshops addressing interactions with the	The steering committee modeled the initiative after Dartmouth College's vertical integration groups (including Dean for Medical Education, Assistant Dean of Curricular Innovation, Pre-Clinical Medical Students Advisory Committee, Clinical Medical Students Advisory Committee, Residents Advisory Committee, Faculty Advisory Committee,	Preclinical and clinical students, and Residents	12.5/18.	14/32.

			pharmaceutical industry and patient-care hand-offs, as well as the development of a tool for patients to evaluate their resident physicians. The establishment of a series of medical education grants aimed at supporting professionalism research has helped raise faculty awareness. This institution's approach and experience to date may provide valuable lessons for educators and leaders aiming to assess and improve their learning environments.	and Assessment and Evaluation Committee)			
35	Iramaneerat [99]	This report summarizes current literature on surgical professionalism to help surgical residency program directors organize appropriate instruction and assessment methods for their residents.	Review of current literature on surgical professionalism	NA	NA	NA	NA
36	Johnston, et al. [115]	Using a novel tool based on General Medical Council (GMC) standards, this cross-sectional survey aimed to assess UK medical students' professional attitudes at different stages of the curriculum, and to investigate the influence of the hidden curriculum on these attitudes through exposure to unprofessional behaviour during the medical course.	An anonymous online questionnaire was developed, reflecting core professional competences outlined in Good Medical Practice. First, third and fifth year students received Section1: 'Attitudes to professionalism'; third and fifth year students also received Section 2: 'Exposure to unprofessional behaviour'. 'Professionalism score' (Section 1) and 'Exposure score' (Section 2) were analysed using ANOVA. Thematic analysis was used to analyse free text.	Medical educators from Queen's University Belfast	Medical students from Queen's University Belfast (First year students were just beginning their training, third year students had completed two years of preclinical training with limited clinical exposure, and fifth year students had completed an additional two years of clinical training.)	10.5/18.	15/32.
37	Kalen, et al. [123]	The aim of this study was to explore Swedish medical students' perceptions of the offered learning activities and their experiences of how these activities were connected to their professional development as defined by the CanMEDS framework.	A prospective mixed method questionnaire study during three terms (internal medicine, scientific project, and surgery) in which data were collected by using contextual activity sampling system, i.e., the students were sent a questionnaire via their mobile phones every third week. All 136 medical students in the 6th of 11 terms in the autumn of 2012 were invited to participate. Seventy-four students (54%) filled in all of the required questionnaires (4 per term) for inclusion, the total number of questionnaires being 1335. The questionnaires focused on the students' experiences of learning activities, especially in relation to the CanMEDS Roles, collaboration with others and emotions (positive, negative, optimal experiences, i.e., "flow") related to the studies. The	Medical Educators from Karolinska Institutet, Sweden	This is a mixed method study and part of a longitudinal study conducted during 2012–2014 at Karolinska Institutet, Sweden, with a focus on undergraduate medical students'	10.5/18.	17/32.

			quantitative data was analysed statistically and, for the open-ended questions, manifest inductive content analysis was used.		professional development.		
38	Kalet, et al. [62]	The authors developed and implemented a program built around a Web-based Professional Development Portfolio (PDP) to assess and document professional development in medical students at New York University School of Medicine.	This program requires students to regularly document their professional development through written reflections on curricular activities spanning preclinical and clinical years. Students post reflections, along with other documents that chronicle their professional growth, to their online PDP. Students meet annually with a faculty mentor to review their portfolios, assess their professional development based on predetermined criteria, and establish goals for the coming year. In this article, the authors describe the development of the PDP and share four years of experience with its implementation. We describe the experiences and attitudes of the first students to participate in this program as reported in an annual student survey. Students' experiences of and satisfaction with the PDP was varied. The PDP has been a catalyst for honest and lively debate concerning the meaning and behavioral manifestations of professionalism. A Web-based PDP promoted self-regulation on an individual level because it facilitated narrative reflection, self-assessment, and goal setting, and it structured mentorship. Therefore, the PDP may prepare students for the self-regulation of the medical profession—a privilege and obligation under the physician's social contract with society.	Medical Educators from New York University School of Medicine	Medical Students	9.5/18.	17/32
39	Katic, et al. [100]	The aim of this study was to explore patient satisfaction with family physicians through evaluation of some characteristics of physician behaviour. The specific goals of this study were to determine whether there were differences in the evaluation of patient satisfaction with physician behaviour with regard to some sociodemographic characteristics of the respondents.	The study group consisted of 1217 respondents: 479 (39.4%) men and 738 (60.6%) women. Medical students interviewed the respondents "face-to-face" immediately after their consultation with the physician. An anonymous questionnaire was created providing answers to 10 questions on patient satisfaction. Data on sociodemographic characteristics and the reason for encounter of the respondents were also collected.	Medical educators from Department of Family Medicine, School of Public Health, Croatia	Medical Students	11/18.	NA
40	Kesselheim, et al. [95]	Educators in pediatric hematology–oncology lack rigorously developed instruments to assess fellows' skills in humanism and professionalism. We developed a novel 15-item self-assessment instrument to address this gap in fellowship training.	We developed a novel 15-item self-assessment instrument to address this gap in fellowship training. Fellows (N=122) were asked to assess their skills in five domains: balancing competing demands of fellowship, caring for the dying patient, confronting depression and burnout, responding to challenging relationships with patients, and practicing humanistic medicine. An expert focus group predefined threshold scores on the instrument that could be used as a cutoff to identify fellows who need support. Reliability and feasibility were assessed and concurrent validity was measured using three established	Medical Educator from Boston Children's Cancer and Blood Disorders Centre	Paediatric Hematology-Oncology Fellows	14.5/18	NA

			instruments: Maslach Burnout Inventory (MBI), Flourishing Scale (FS), and Jefferson Scale of Physician Empathy (JSPE).				
41	Malakoff, et al. [114]	We sought a means to objectively assess professionalism among internal medicine and transitional year residents.	We established a point system to document unprofessional behaviors demonstrated by internal medicine and transitional year residents along with opportunities to redeem such negative points by deliberate positive professional acts. The intent of the policy is to assist residents in becoming aware of what constitutes unprofessional behavior and to provide opportunities for remediation by accruing positive points. A committee of core faculty and department leadership including the program director and clinic nurse manager determines professionalism points assigned. Negative points might be awarded for tardiness to mandatory or volunteered for events without a valid excuse, late evaluations or other paperwork required by the department, non-attendance at meetings prepaid by the department, and inappropriate use of personal days or leave. Examples of actions through which positive points can be gained to erase negative points include delivery of a mentored pre-conference talk, noon conference, medical student case/shelf review session, or a written reflection.	Medical Educators from University of Tennessee College of Medicine	Internal medicine and transitional year residents	11/18.	NA
42	Menna, et al. [85]	The College of Medicine at the University of Arkansas for Medical Sciences (UAMS) has developed an easy and practical method of evaluating medical student professionalism.	The evaluation instrument is a single page document listing parameters of professionalism. Next to each parameter are the options of designating a student as either "Inadequate" or "Outstanding" i.e. is the student unable to meet or has he/she exceeded the College's expectations of professional behavior. The form also provides space for comments from the evaluator. A comment is required for all "Inadequate" ratings; "Outstanding" ratings do not require comments but are strongly encouraged. The process allows for faculty, nurses, residents, clerical staff, and even other medical students to submit a form. Generally, however, faculty is the major source of these reports. The use of this form greatly facilitates the evaluation of medical student professionalism and importantly saves faculty time.	Medical Educators from the College of Medicine at the University of Arkansas for Medical Sciences (UAMS)	Medical students	11/18.	NA
43	Nagler, et al. [22]	To understand these differences (in perceptions of professional behavior may vary by individual, medical specialty, demographic group and institution) should help institutions better clarify professionalism expectations and provide standards with which to evaluate resident behavior	Duke University Hospital and Vidant Medical Center/East Carolina University surveyed entering PGY1 residents. Residents were queried on two issues: their perception of the professionalism of 46 specific behaviors related to training and patient care; and their own participation in those specified behaviors. The study reports data analyses for gender and institution based	Duke University Hospital and Vidant Medical Center/East Carolina University	PGY1 residents (DUH had over 950 residents training in one of 77 ACGME programs, and 60 internally	12/18.	13/32.

			upon survey results in 2009 and 2010. The study received approval by the Institutional Review Boards of both institutions.		sponsored programs. VMC/ECU had 340 residents training in one of 28 ACGME programs, and 5 internally sponsored programs.)		
44	Olsson, et al. [98]	To determine the internal consistency and the underlying components of our translated and adapted Swedish version of the General Medical Council's multi source feedback questionnaires (GMC questionnaires) for physicians and to confirm which aspects of good medical practice the latent variable structure reflected.	From October 2015 to March 2016, residents in family medicine in Sweden were invited to participate in the study and to use the Swedish version to perform self-evaluations and acquire feedback from both their patients and colleagues. The validation focused on internal con-sistency and construct validity. Main outcome measures were Cronbach's alpha coefficients, Principal Component Analysis, and Confirmatory Factor Analysis indices.	Medical Educators from Division of Family Medicine, Karolinska Institutet, Stockholm, Sweden	Family Medicine Residents	16/18.	NA
45	Ortwein, et al. [103]	In order to evaluate the validity of this catalogue, we surveyed anaesthetists at our department in regard to their perception the importance of each of these items. In addition to the descriptive acquisition of data, it was intended to assess the results of the survey to ascertain whether there were differences in the evaluation of these objectives by specialists and registrars.	The questionnaire with the seven adapted CanMEDS Roles included items describing each of their underlying competencies. Each anaesthetist (registrars and specialists) working at our institution in May of 2007 was asked to participate in the survey. Individual perception of relevance was rated for each item on a scale similar to the Likert system, ranging from 1 (highly relevant) to 5 (not at all relevant), from which ratings means were calculated. For determination of reliability, we calculated Cronbach's alpha. To assess differences between subgroups, we performed analysis of variance.	Medical Educators from Department of Anaesthesiology and Intensive Care Medicine, and Institute for Educational Progress, Universitat zu Berlin, Germany	Specialist and registrar	10.5/18.	NA
46	O'Sullivan, Toohey [131]	The aim was to determine whether specific aspects of professionalism were underdeveloped in medical students.	A questionnaire with 24 vignettes was taken by Year 2, 4, and 6 medical students and their responses were compared to responses from practicing Medical Academics.	Medical educators from Department of Medicine, St. George Hospital	Year 2, 4, 6 medical students, and practising Medical Academics	10/18.	NA
47	Pavon, et al. [118]	The authors developed a Transitions of Care (TOC) curriculum to teach and measure learner competence in performing TOC tasks for older adults.	Internal medicine interns at an academic residency program received the curriculum, which consisted of experiential learning, self-study, and small group discussion. Interns completed retrospective pre/post surveys rating their confidence in performing five TOC tasks, qualitative open-ended survey questions, and a selfreflection essay. A subset of interns also completed follow-up assessments.	Medical educators from Duke University Medical Centre	Internal Medicine and Family Medicine Medical residents	13.5/18.	17/32.

48	Peterkin, et al. [122]	The purpose of this pilot project was to determine if using reflective writing in teaching the CanMEDS roles helps to increase students' understanding of the roles in the clinical context.	A pilot project was undertaken with 10 third-year medical students at the University of Toronto in Ontario. Students wrote about a different CanMEDS role for each session based on supplied writing prompts. Students also completed a Narrative Reflection Tool at the end of each group session. A selection of writing samples was assessed for reflection and for an understanding of the CanMEDS roles. Students were also given an opportunity to provide feedback on the program.	Medical Educators from University of Toronto in Ontario	Third Year Medical Students	NA	15/32.
49	Peterson, et al. [94]	Given the novelty of those observations, the purpose of this study was to test their generalizability by evaluating an MD program as a whole.	The Readiness for Residency Survey (RfR) was developed and aligned with the published Readiness for Clerkship Survey (RfC), but focused on the competencies expected to be achieved at graduation. The RfC and RfR were administered electronically four months after the start of clerkship and six months after the start of residency, respectively. Generalizability and decision studies examined the extent to which specific competencies were achieved relative to one another.	Medical Educators from Evaluation Studies Unit, Faculty of Medicine, Diamond Health Science Centre, Canada	Residents in the disciplines of family medicine, internal medicine, pediatrics, psychiatry, obstetrics–gynecology, or general surgery	12.5/18.	NA
50	Picho, et al. [84]	This study assessed alumni perceptions of their preparedness for clinical practice using the Accreditation Council for Graduate Medical Education (ACGME) competencies.	1,189 alumni who graduated from the Uniformed Services University (USU) between 1980 and 2001 completed a survey modeled to assess the ACGME competencies on a 5-point, Likert-type scale. Specifically, self-reports of competencies related to patient care, communication and interpersonal skills, medical knowledge, professionalism, systems-based practice, practice-based learning and improvement, and militaryunique practice were evaluated.	Medical Educators from Department of Medicine, Uniformed Services University of Health Science	Graduates from the school	10.5/18.	NA
51	Rademakers, et al. [129]	As changes in postgraduate training will probably be most effective if future trainees recognise their value, we set out to determine how senior medical students rated these fields of competency in terms of their importance.	We carried out a study at University Medical Centre (UMC) Utrecht, the Netherlands, in which 80 Year 6 medical students answered a questionnaire in which they rated the importance of each of 28 key competencies within the 7 competency fields.	Medical Educators at Centre for Research and Development of Education, School of Medical Sciences, University Medical Centre, Utrecht, Netherlands	Year 6 Medical Students	10.5/18.	NA
52	Raee, et al. [109]	The aim of this study is to evaluate the professional behavior and performance in medical students in the form of team based assessment.	In a cross-sectional study, 100 medical students in the 7th year of education were randomly selected and enrolled; for each student five questionnaires were filled out, including one self-assessment, two peer assessments and two residents assessment. The scoring system of the questionnaires was based on seven point Likert scale. After filling out the questions in the questionnaire, numerical data and written comments provided to the students were collected, analyzed and discussed. Internal	Self-assessment, Peer-assessment, Resident assessment	Final year Medical Students	14/18.	NA

			consistency (Cronbach's alpha) of the questionnaires was assessed. A p<0.05 was considered as significant.				
53	Rawlings, et al. [92]	This study demonstrates the process for developing and evaluating narrative cases representing the five levels of the professionalism milestones.	In 2013, the authors identified 28 behaviors in the Accreditation Council for Graduate Medical Education general surgery professionalism milestones. They modified previously published narrative cases to fit these behaviors. To evaluate the quality of these cases, the authors developed a 28-item, five-point scale instrument, which 29 interdisciplinary faculty completed. The authors compared the faculty ratings by narrative case and specialty with the authors' initial rankings of the cases by milestone level. They used t tests and analysis of variance to compare mean scores across specialties.	Medical educators from University of Missouri School of Medicine	Residents from University of Missouri School of Medicine	14/18.	NA
54	Roberts, et al. [125]	We sought to learn which unprofessional behaviors were endemic in our school, and which were unique to particular departments.	Students graduating from medical school between 2007 and 2012 were asked to complete a questionnaire naming the most professional and least professional faculty members they encountered in during school. For the least professional faculty members, they were also asked to provide information about the unprofessional behavior.	Final year medical students	Faculty members	7/18.	13/32.
55	Roberts, et al. [106]	To increaseorthopaedicsurgeryresident understandingoftheuniqueneedsofolderadultsinorder to maintaineffectiveandsensitivecommunicationwiththis vulnerablepopulation.	A two-parttrainingprogram(ongoingfor8 years)comprisedof:1)small-groupinteractivedidactic sessionsonagingissues;and2)workshopdemonstrations given bytheresidentstoagroupofolderadults,followedby a Question&Answersession.Residentswereassessedusing a 22-itempre–postquestionnairecoveringmedicalknowl-edge ofaging,attitudestowardolderadults,andpersonal anxietyaboutaging.Olderadultparticipantsweresurveyed for perceptionsofresidents' sensitivitytowardthem.	Self	Orthopedic surgery residents	13.5/18.	NA
56	Rodriguez, et al. [132]	One goal of the 2012 Academic Emergency Medicine consensus conference on education research in EM was to develop a research agenda for testing and developing tools to assess professionalism in EM residents.	Prior to the consensus conference, a comprehensive review of the published literature on professionalism assessment was performed. The Medline database was searched using the terms "professionalism" and "humanism" independently combined with the term "assessment," resulting in 464 and 211 references, respectively. Combining "professionalism" and "evaluation" identified 509 references.	Self, peers, nurses, faculty, and/or patients	Emergency Medicine Residents	NA	NA
57	Sang, et al. [117]	This study assessed the initial validity and reliability of the Vietnamese Physician Professional Values Scale (VPPVS).	A sample of clinical experts reviewed the VPPVS to ensure face and content validity of the scale, resulting in a draft 37-item measure. A cross-sectional survey of 1086 physicians from Hanoi, Hue and Ho Chi Minh City completed a self-report survey, which included the draft of the VPPVS. Exploratory Factor Analysis was used to assess construct validity, resulting in 35 items assessing	Medical Educators from Centre for Research and Training on HIV/AIDS, Hanoi Medical University, Hanoi, Vietnam	Vietnamese Physician (specialist areas of paediatrics, dentistry, traditional medicine and	11/18.	NA

			physician's professional values across five main factors: lifestyle, professionalism, prestige, management and finance. The final five-factor scale illustrated acceptable internal consistency, with Cronbach's alpha coefficients ranging from 0.73 to 0.86 and all item-total correlations >0.2. Limited floor or ceiling effects were found.		obstetrics and gynaecology)		
58	Santen, et al. [102]	The objective of this study was to develop competencybased milestones for fourth-year medical students completing their emergency medicine (EM) clerkships (regardless of whether the students were planning on entering EM) using a rigorous method to attain validity evidence.	A literature review was performed to develop a list of potential milestones. An expert panel, which included a medical student and 23 faculty members (four program directors, 16 clerkship directors, and five assistant deans) from 19 different institutions, came to consensus on these milestones through two rounds of a modified Delphi protocol. The Delphi technique builds content validity and is an accepted method to develop consensus by eliciting expert opinions through multiple rounds of questionnaires.	Medical Educators from Department of Emergency Medicine, University of Michigan Medical School	Fourth year medical student	NA	NA
59	Santosa, et al. [124]	The objective of this study was to develop an instrument to assess professionalism for psychiatric residents adapted from ACGME (Accreditation Council for Graduate Medical Education).	This study was a quantitative non-experimental study consisted of 3 stages (literature review, peer review, and validity and reliability testing). The subjects were residents on training at the Department of Psychiatry, Medical Faculty, Gadjah Mada University. The construct validity testing was conducted with Confimatory Factor Analysis (CFA) and content validity with Pearson's product moment. Reliability testing was conducted with Cronbach's alpha.	Medical Educators from Department of Psychiatry, Faculty of Medicine, Universitas Gadjah Mada - Indonesia	Psychiatry Residents	15/18.	NA
60	Stockley, Forbes [126]	This study sought to explore the students' experiences of these tutorials in order to develop the evidence base further.	Sixteen medical students participated in three focus-group interviews exploring their experiences of medical professionalism tutorials. A course evaluation questionnaire to all fifth year students also provided data. Data were analysed using the principles of Interpretative Phenomenological Analysis.	University of Bristol	Fifth Year Medical Students	NA	22/32.
61	Strowd, et al. [83]	A cross-sectional study was performed to describe perceptions of professionalism among representative physicians across the academic training spectrum of neurology.	A self-report questionnaire adapted from a published instrument was distributed to students, residents, fellows, and faculty in neurology at a single institution. Responders rated 4 domains of professionalism: Personal Characteristics, Interactions with Patients, Social Responsibility, and Interactions with the Health Care Team (5-point Likert scale, not at all important to very important), and selected the "top 2" characteristics critical to professional behavior in each domain.	Medical Educators from Department of Neurology, Johns Hopkins Hospital, Baltimore	A cross-sectional study was designed consisting of medical students, residents, clinical fellows, and faculty physicians in the department of neurology at a single academic institution.	11/18.	NA

62	Sullivan, et al. [116]	The purpose of this study is to report survey results regarding the identification and rating of unprofessional behaviors and challenges in the evaluation and remediation of professionalism.	In June 2010, the task force sent an anonymous survey via the CORD-EM listserv to PDs with active EM programs.	Medical Educators from Department of Emergency Medicine, University of Missouri-Kansas City of School of Medicine, Trauma Medical Centre	Programme Directors from Emergency Medicine	7.5/18.	NA
63	Tanaka, et al. [86]	The goals of this study were to (1) conduct focus groups of anesthesia residents to define what constitutes optimal feedback; (2) develop, test, and implement a web-based feedback tool; and (3) then map the contents of the written comments collected on the feedback tool to the Accreditation Council for Graduate Medical Education (ACGME) anesthesiology milestones.	All 72 anesthesia residents in the program were invited to participate in 1 of 5 focus groups scheduled over a 2-month period. Thirty-seven (51%) participated in the focus groups and completed a written survey on previous feedback experiences. On the basis of the focus group input, an initial online feedback tool was pilot-tested with 20 residents and 62 feedback sessions, and then a final feedback tool was deployed to the entire residency to facilitate the feedback process. The completed feedback written entries were mapped onto the 25 ACGME anesthesiology milestones.	Medical Educators from Department of Anesthesiology, Perioperative and Pain Medicine, Stanford University School of Medicine, Stanford, California	Anaesthesia Residents	12/18.	17/32.
64	Taylor, et al. [111]	To review and assess educational strategies and formats regarding communication with families/survivors in the aftermath of unexpected and untimely patient death. To propose an integrated curriculum designed and intended to foster proficiency, competence, confidence, and composure in relaying catastrophic information in the context of the professional experience of a cohort of seasoned surgeons.	Survey of a cohort of senior surgeons (membership of the Southern Surgical Association) and Surgical Residency Program Directors (membership of the Association of Program Directors in Surgery).	Medical Educators from Department of Surgery, College of Medicine and Medical Center, University of South Alabama, Mobile, Alabama	Senior surgeons (membership of the Southern Surgical Association) and Surgical Residency Program Directors (membership of the Association of Program Directors in Surgery)	7/18.	NA
65	Todhunter, et al. [97]	The immediate objective of this study is to develop and pilot such an instrument to permit students to assess the professionalism of their clinical teachers.	Part I: development of the faculty professionalism form When developing an evaluation tool, it is important that the tool is acceptable to all users. Therefore, both students and faculty within the McGill community were an important part of the reported development process. Part II: pilot testing of the faculty professionalism evaluation form With the collaborative process of development, and the inclusion of both student and faculty stakeholders in the development of the Faculty Professionalism Evaluation Form, a small-scale piloting of the form was undertaken.	Students from McGill Community	Faculty members from McGill	11/18.	11/32.

66	Tsai, et al. [20]	The main purpose of this study was to identify and understand the structure of latent traits underlying the concept of medical professionalism of Taiwanese students.	A 32 item questionnaire assessing medical professionalism derived from the definition by the American Board Internal Medicine (ABIM) was distributed to 133 year seven medical students. A five-point rating scale of importance was used to identify the extent of their values or beliefs in each item.	Meducal Educators from College of Medicine National Cheng-Kung University, Taiwan	7th Year Medical Students	9.5/18	NA
67	van de Camp, et al. [78]	The aim of this study is to develop a new tool to assess professional behaviour in general practitioner (GP) trainees: the evaluation of professional behaviour in general practice (EPRO-GP) instrument.	Our study consisted of 4 phases: (1) development of a model of professionalism in general practice based on a literature review on professionalism, competency models of general practice and the overall educational objectives of postgraduate training for general practice; (2) development of the EPRO-GP instrument in collaboration with a sounding board; (3) establishing the content validity of the EPRO-GP instrument using a nominal group technique; and (4) establishing the feasibility of the EPRO-GP instrument in 12 general practice trainees and their general practice trainers.	General Practice Trainer	General Practice Trainee	13/18.	10/32.
68	Vora, et al. [107]	This project aimed to pilot an innovative SP-based model to assess the interpersonal communication skills and professionalism Milestones of emergency medicine (EM) residents.	This paper outlines an innovative method of assessing these Milestones using an established instructional method. EM faculty mapped the communication and professional values Milestones to an existing communication and interpersonal skills scale. We identified six communication-focused scenarios: death notification; informed consent; medical non-compliance; medical error; treatment refusal; and advanced directives. In a pilot, 18 EM residents completed these six standardized patient (SP) encounters. Our experience suggests SP encounters can support standardized direct observation of residents' achievement of ACGME Milestones.	University of Illinois-College of Medicine at Chicago Clinical Performance Centre (Each SP encounter was 10 minutes, with the SP completing the RUCIS scale immediately following the encounter. This was followed by 10 minutes for SP-to-resident debriefing.)	Emergency Medicine Residents	14/18.	NA
69	Mak-van der Vossen, et al. [113]	To identify patterns in the unprofessional behaviors of medical students and to construct descriptions based on these patterns.	Content analysis of research articles yielded a template of unprofessional behaviors for coding student evaluation forms indicating unsatisfactory professional behavior, collected from 2012 to 2014 at the VUmc School of Medical Sciences, Amsterdam, the Netherlands. Latent class analysis was used to identify classes of students with a high chance of displaying comparable unprofessional behaviors. Teachers' feedback of prototype students was summarized to generate profile descriptions.	The study was conducted at VUmc School of Medical Sciences, Amsterdam, the Netherlands.	The derived sample consisted of 232 evaluation forms from students with unsatisfactory professional behavior (120 forms of 89 preclinical undergraduate students and 112 forms of 105 clinical undergraduate students),	NA	15/32.

					representing 7.9% of 2,460 students (3.9% per year). Twenty-seven students (1.1% of total student population) received multiple unsatisfactory professional behavior evaluations.		
70	Warren, et al. [80]	To (a) understand what program directors felt were key elements of the CanMEDS Professional Role and (b) identify the teaching and assessment methods they used.	A two-step sequential mixed method design using a survey and semi-structured interviews with Canadian program directors.	Candian program directors from Dalhousie University	Our sample included Canadian post-graduate residency program directors in the seven largest RCPSC specialties (i.e. Anesthesiology, Diagnostic Radiology, General Surgery, Internal Medicine, Obstetrics & Gynecology, Pediatrics and Psychiatry).	13/18.	18/32.
71	Wilkinson, et al. [4]	This study had four aims: • To synthesize the various definitions and interpretations of professionalism • To describe a toolbox of possible assessment methods • To produce a blueprint that matches assessment tools to the identified elements of professionalism • To identify gaps where professionalism elements are not well matched by assessment tools	of definitions of professionalism and of relevant assessment tools, clustered the definitions of professionalism into assessable components, and clustered assessment tools of a similar nature. They then created a "blueprint" whereby the elements of professionalism are matched to relevant assessment tools.	NA	NA	NA	NA
72	Williams, et al. [79]	The purpose of this study was to determine the degree of convergence of two measurement methods, one based on Miller's framework, the second using the Accreditation Council for Graduate Medical Education/American Board of Medical Specialties (ACGME/ ABMS) Core Competency framework.	The purpose of this study was to determine the degree of convergence of two measurement methods, one based on Miller's framework, the second using the Accreditation Council for Graduate Medical Education/American Board of Medical Specialties (ACGME/ ABMS) Core Competency framework. The data were gathered from the faculty of a large, Midwestern regional health care provider and hospital system. Data from 264 respondents	Raters of physicians holding supervisory roles in the organization (both self-assessment and rater-assessment)	Physicians from the faculty of a large, Midwestern regional health care provider and hospital system	16/18.	NA

			were studied. The 360 data were from raters of physicians holding supervisory roles in the organization. The scale items were taken from an instrument that has been validated for both structure and known group prediction.				
73	Williams, et al. [110]	The purpose of this study was to determine agreement between these two assessment methodologies with the goal of finding a more efficient and less burdensome method of assessing physician competence in the workplace.	Three hundred sixty-degree data were collected from 264 raters of physicians holding supervisory roles. Raters included the physicians' leaders, peers, and support/reports. The scale items were taken from an instrument developed for the assessment of interpersonal and communications skills, professionalism, and system-based practice. The Dreyfus scale was purposely built for this application.	264 raters of physicians holding supervisory roles. Raters included the physicians' leaders, peers, and support/reports.	The data were gathered from the faculty of a large Midwestern regional health care provider and hospital system (each faculty ember was assessed both by others and themselves (360 deg survey).	14.5/18.	NA
74	Yazdankhah, et al. [91]	This study was conducted to assess professionalism in surgical residents, using a 360-degree evaluation technique in several teaching hospitals in Tehran, Iran.	This study was conducted on all the second and third year surgery residents from three university teaching hospitals in Tehran. Multi-source feedback questionnaire contained 10 questions on the residents' professional behavior and was completed by the faculty and staff members (nurses, operation room staff, and medical assistants) as well as other surgery residents, interns and patients to evaluate each resident. Response rates were used to determine feasibility for each of the respondent groups and the mean and standard deviation score for each question was computed to determine the viability of the items. Reliability was assessed using alpha Cronbach coefficient for each respondent group. The correlation between these scores and the residents' final and OSCE grade was also assessed.	Every surgery resident was evaluated by the faculty and staff members (nurses, operation room staff, and medical assistants) as well as other surgery residents, interns and patients with whom they worked regularly during the past three months. (from three university teaching hospitals in Tehran, all affiliated to Tehran University of Medical Sciences)	All the 2nd and 3rd year surgery residents from these hospitals were required to participate in the process.	12.5/18.	NA
